# Novel Coumarin-Substituted Cyclophosphazene as a Fluorescent Probe for Highly Selective Detection of 2,4,6-Trinitrophenol

**DOI:** 10.1021/acsomega.4c05306

**Published:** 2025-02-03

**Authors:** Ishanki Sharma, Rajeev Kumar Sinha, Suranjan Shil, Shruti Rani, N. V. Anil Kumar

**Affiliations:** † Department of Chemistry, Manipal Institute of Technology, Manipal Academy of Higher Education, Manipal 576104, India; ‡ Department of Physics, Birla Institute of Technology Mesra, Ranchi 835215, India; § Manipal Centre for Natural Sciences, Manipal Academy of Higher Education, Manipal 576104, India; ∥ Department of Chemical Sciences, Indian Institute of Science, Education and Research (IISER), Mohali 140306, India

## Abstract

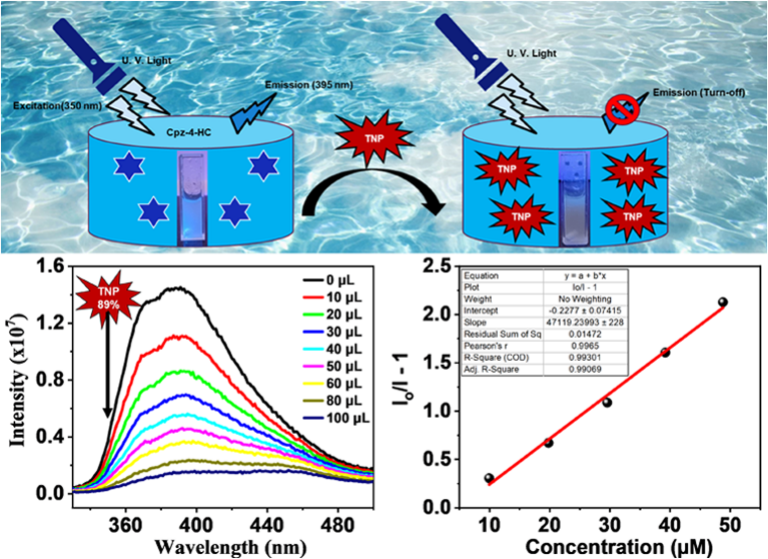

Nitroaromatic compounds (NACs) such as 2,4,6-trinitrophenol (TNP), commonly known as picric acid (PA), hold widespread application in industries such as dyestuff production, wood preservation, explosives manufacturing, insect control, and photographic development. In this study, the organic–inorganic hybrid 4,4′,4″,4‴,4″″,4‴″-((1,3,5,2λ5,4λ5,6λ5-triazatriphosphinine-2,2,4,4,6,6-hexayl)hexakis(oxy))hexakis(2*H*-chromen-2-one) (**Cpz-4-HC**) was synthesized via the nucleophilic substitution reaction of 4-hydroxycoumarin (4-HC) with hexachlorocyclotriphosphazene (HCCP). The structure of Cpz-4-HC was fully characterized by Fourier transform infrared (FT-IR), ^1^H-, ^13^C-, and ^31^P NMR, and HRMS. **Cpz-4-HC** is used as a chemical fluorescence sensor for the detection of TNP, with a *K*_SV_ value of 4.71 × 10^4^ M^–1^ and a low limit of detection (LOD) of 0.334 ppm over some other analytes such as 2,4-DNP, 4-NP, 2-NP, 1,3-DNB, 2,4-DNT, and 2,6-DNT in water. The sensing mechanism was elucidated through spectral overlap analysis, indicating the resonance energy transfer as the dominant quenching process. Dynamic quenching was established through fluorescence lifetime studies, further affirming **Cpz-4-HC** capability for environmental monitoring. Experimental and theoretical analyses underscored TNP’s strong interaction with **Cpz-4-HC**, corroborating its suitability for sensing applications. Their recyclable nature and ultrafast response time make them highly suitable for detecting TNP, even in the presence of other interfering nitroaromatics. This study provides novel perspectives on the development and formulation of a chemical fluorescent sensor for TNP, utilizing a straightforward synthesis method.

## Introduction

Nitroaromatic compounds (NACs) play a crucial role in several industries, such as petroleum, gas, textiles, medicines, wood, papermaking, insecticides, and dyestuffs. Functioning as crucial catalysts in chemical reactions, they contribute to the generation of byproducts such as nitro-toluene isomers.^1^ Among their many uses, NACs are found in wastewater, rivers, and soil treated with herbicides or pesticides. These compounds are also used to make dyes, plastics, explosives, herbicides, and pesticides. These substances possess the potential to harm living organisms and can also give rise to environmental and security issues. Mononitrophenols and dinitrophenols have been designated as priority pollutants by the US Environmental Protection Agency because of their significant toxicity.^2,3^

Picric acid (PA), alternatively termed 2,4,6-trinitrophenol (TNP), holds widespread application in industries such as dyestuff production, pharmacy, and leather processing. Recognized for its phenolic and nitro functionalities, PA poses known hazards to living systems.^4^ In addition to its chemical qualities, the fact that PA is a nitroaromatic compound renders it very explosive, which explains its application in incendiaries, rocket fuels, pyrotechnics, and matches. TNP mishandling or inappropriate disposal not only endangers the environment and has a detrimental impact on human health but also raises issues about public safety. Therefore, it is imperative to explore methods that are more convenient, sensitive, cost-effective, operationally easy, and selective to detect TNP.

Recently, various techniques have been adopted to detect explosives, including gas chromatography–mass spectrometry (GC-MS), surface-enhanced Raman spectroscopy, electrochemical methods, X-ray spectrometry, high-performance liquid chromatography, and mass spectrometry.^5−7^ These methods are primarily based on field sampling.^8^ The current methods face challenges, including time consumption, operational complexity, and high costs, limiting their on-site applicability.^5^ Fluorescent probes have a promising option in recent years due to distinctive advantages, including real-time monitoring, nonseparation, cost-effectiveness, ease of operation, and visual detection.^9^ These sensors work either by fluorescence quenching or turn-on when they bind with nitro-substituted aromatic compounds.^10^ To date, a myriad of fluorescent chemical sensors targeting nitroaromatics has emerged, utilizing diverse fluorescent dye molecules, electron donor conjugated polymers, metal–organic framework, quantum dot semiconductor nanocrystals, and fluorescent organic/inorganic nanohybrids. Despite these advancements, the applicability of these sensing systems in picric acid (PA) detection is hindered by challenges such as interference from structurally similar nitroaromatics, complex synthesis routes for conjugated polymers, and the potential environmental and health risks associated with certain sensor components.^11^

Coumarin derivatives are currently regarded as highly attractive fluorophore groups due to their extensive research and applications, attributed to their high molar absorption coefficient, large Stokes shift, elevated fluorescence quantum yield, and notable physiological activity. This suggests that coumarin holds the potential as a preferred fluorophore for the development of fluorescent chemosensors.^12^ 4-Hydroxycoumarin has attracted considerable interest due to its significant UV absorption at 308 nm and energy gap of about 3.78 eV, and its donating nature facilitated by the −OH group. Its exceptional fluorescent properties make it highly suitable for application as fluorescent markers, dyes, and stains. It is extensively utilized due to its high emission yields, photostability, extended spectral range, and excellent solubility.^13^ Since the cyclotriphosphazene scaffold, in contrast, lacks inherent fluorescent properties but serves as a versatile platform for the synthesis of molecules with different and tunable properties depending on the varying degree of substitution.^14^

Tümay and Yeşilot et al. introduced an innovative fluorescent sensor utilizing a water-soluble, double-anthracene-bridged cyclotriphosphazene.^15^ This sensor demonstrates ultrasensitivity and high selectivity in spectrofluorometric determining TNT in environmental waters. Tümay and Yeşilot et al. have chosen this molecule as a sensor due to its outstanding features, which include great thermal stability and increased susceptibility to nucleophilic reactions in basic circumstances.^15^ Utilizing hexachlorocyclotriphosphazene (NPCl_2_)_3_ as a core, the researchers crafted various multifunctional materials, as well as devices for advanced technology. Furthermore, the inclusion of modifying groups on phosphorus improved the optical properties of cyclotriphosphazene, addressing the optical inertness commonly reported in cyclophosphazenes in the ultraviolet–visible (UV–vis) spectrum. This optical feature is critical in the recent advancements of cyclotriphosphazene-based sensors, exhibiting intramolecular or intermolecular CH–π and π–π stacking interactions.^16^ The investigation of fluorescent methods for TNP detection remains a compelling challenge. While numerous fluorescent sensors can effectively detect TNP in nonaqueous mediums, challenges arise in water-containing environments due to potential interference.

To overcome challenges in TNP probes, such as limited Stokes shift^9,16^and wavelength emission constraints causing issues like FRET^9^ and photon reabsorption,^9^ a novel approach that integrates two strategies in a single molecular probe. This aims to not only broaden the Stokes shift but also boost quantum yield, thereby enhancing detection sensitivity in multiplex biodetection.^9^ We successfully synthesized a coumarin derivative Cpz-4-HC, which exhibited outstanding fluorescence emission at 395 nm. The chemical structure of Cpz-4-HC was thoroughly characterized using Fourier-transform infrared (FT-IR), nuclear magnetic resonance (NMR), and high-resolution mass spectrometry (HRMS). In our research, we combined hexachlorocyclotriphosphazene (HCCP) and coumarin to effortlessly create a fluorescent chemosensor for various tested NACs in solution. The study investigates the fluorescence sensing capabilities of Cpz-4-HC toward NACs, particularly focusing on its ability to detect NACs in water. Through fluorescence quenching experiments and spectral analysis, Cpz-4-HC demonstrates high sensitivity and selectivity toward TNP over other NACs. The quenching mechanism, elucidated through fluorescence lifetime studies and computational modeling, suggests a dynamic quenching process driven by electron transfer, particularly evident in the case of TNP due to its strong H-bonding interaction with Cpz-4-HC. These findings underscore Cpz-4-HC as a promising candidate for environmental monitoring applications, offering insights into its detection mechanism and potential for selectively sensing nitrophenols in aqueous environments. The effectiveness of Cpz-4-HC for real-time applications was validated using a paper strip detection test and recyclability experiment.

## Results and Discussion

### Synthesis and Characterization of 4-Hydroxycoumarin-Substituted Cyclotriphosphazene Cpz-4-HC

**Scheme 1:**
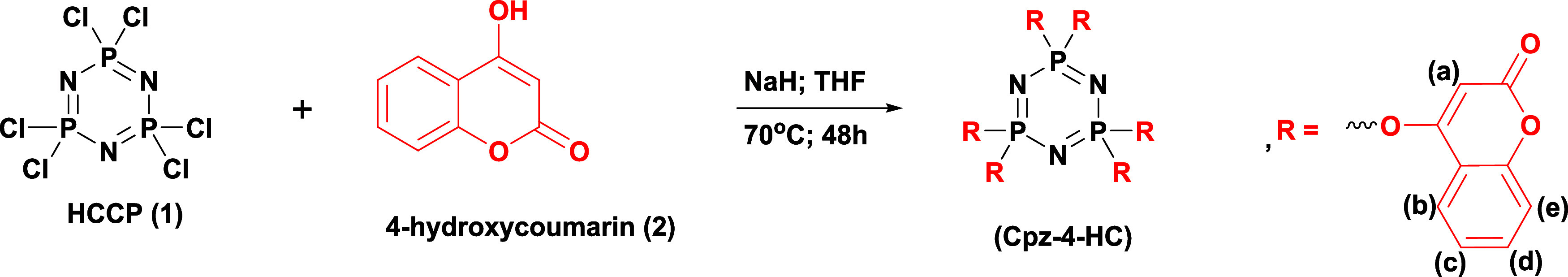
Synthetic Route and Proposed Chemical Structure

In this research, we synthesized 4,4′,4″,4‴,4″″,4‴″-((1,3,5,2λ^5^,4λ^5^,6λ^5^-triazatriphosphinine-2,2,4,4,6,6-hexayl)hexakis(oxy))hexakis(2*H*-chromen-2-one) (Cpz-4-HC) through a straightforward, facile, and one-step single displacement reaction of hexachlorocyclotriphosphazene and 4-hydroxycoumarin. The process involves the reaction between hexachlorocyclotriphosphazene (1) and 4-hydroxycoumarin (2), yielding derivatives of cyclotriphosphazene with six substitutions of the coumarin moiety, as depicted in Scheme 1. To obtain pure products, a combination of column chromatography and preparative TLC was employed for purification purposes. The chemical structures of the isolated substances were analyzed using various techniques, including HRMS mass spectrometry (Figure S5), FT-IR spectroscopy, as well as ^1^H, ^13^C, and ^31^P NMR spectroscopy. Figure S1 depicts the structural analysis of Cpz-4-HC using FT-IR measurements.

When looking at the FT-IR spectrum of the compound, Cpz-4-HC showed characteristic stretching bands for aromatic CH at 2672 cm^–1^, and a sharp peak was observed for C=O vibrations at 1728 cm^–1^. The vibration bands assignable to the stretching of the P=N bands were observed at a frequency of 1222 cm–1, while P–N was observed around 772 cm^–1^. The distinctive vibrations associated with ether groups (C–O–C) were also noted at 1127 cm^–1^. According to the peak attributions described above, Cpz-4-HC contains both HCCP and 4-HC structural units. Furthermore, the spectra of Cpz-4-HC reveal the absence of the absorption peak corresponding to the phenolic hydroxyl groups of 4-HC at 3518 cm^–1^. Simultaneously, a new absorption peak at 1079 cm^–1^ emerges in the Cpz-4-HC spectra, indicating the formation of P–O–C bonds. This finding confirms that the synthesis of Cpz-4-HC resulted from a nucleophilic substitution reaction between HCCP (1) and 4-HC (2), where NaH acts as a strong base, facilitating the deprotonation of the hydroxyl group. When examining the proton NMR spectrum of hexasubstituted Cpz-4-HC, it was observed that the −OH proton was replaced by a chlorine atom bonded to the phosphorus atom. This occurrence serves as a crucial indicator of structural modification. The aromatic protons were detected within the range of 7.64 to 6.53 ppm, while the aromatic carbons exhibited signals in the range of 173.75 to 106.92 ppm. The ^31^P NMR spectra revealed a single signal, confirming full replacement. The similar chemical shift at 5.64 ppm demonstrates that all phosphorus atoms have a consistent chemical environment. This resonance pattern, marked by a single sharp peak, represents the phosphorus nuclei’s equivalency, generating an A3 spin system inside the molecule. The ^1^H, ^13^C, and ^31^P NMR spectra are presented in Section S-1 and Figures S2–S4.

#### SEM

**Figure 1:**
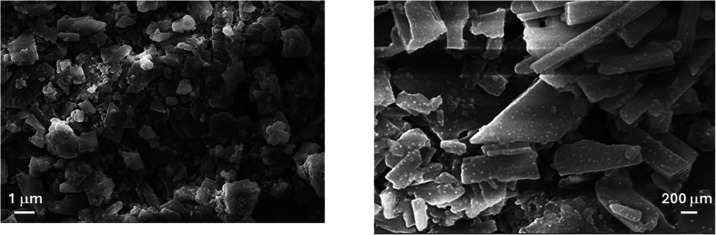
SEM of Cpz-4-HC at x50,000 magnification.

Scanning electron microscopy (SEM) was utilized to analyze the topographical and morphological features of Cpz-4-HC. The SEM micrographs of Cpz-4-HC, presented in Figure 1, reveal aggregated, flake-like crystalline structures with flat surfaces and sharp edges. The average particle size calculated was around 0.893 μm.

### Thermal Stability

**Figure 2:**
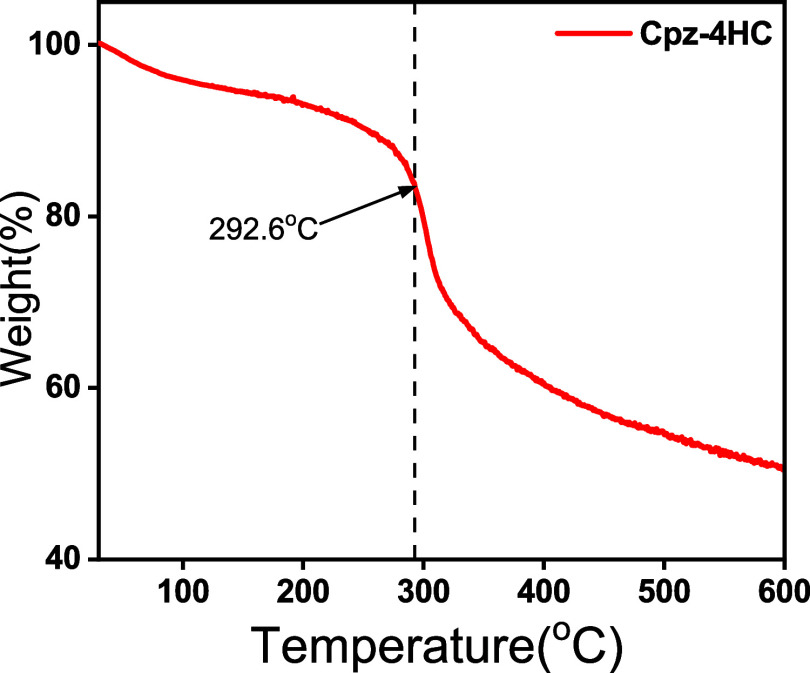
TGA curve of Cpz-4-HC.

TGA of Cpz-4-HC was performed with a temperature range from 30 to 600 °C, with a heating rate of 10 °C min^–1^. The thermal decomposition temperature of Cpz-4-HC was observed at 292.6 °C (Figure 2), indicating excellent thermal stability. This is consistent with the melting point of Cpz-4-HC, which was observed at 292.9 °C.

### Photophysical Properties

The photophysical properties of novel hexa-4-hydroxycoumarin-substituted cyclotriphosphazene were evaluated by solid-state UV–vis and fluorescence spectroscopy (Section S-1; Figures S6 and S7). The absorption spectra of Cpz-4-HC in its solid-state display peak absorption values at approximately 283 and 311 nm, which are associated with π–π* and n−π* transitions, respectively. The absorption properties can be ascribed to transitions between π and π* orbitals of 4-hydroxycoumarin fluorophore.^17^ Due to their optical inertness in the UV–vis region, phosphazene compounds can have their optical properties adjusted by incorporating specific fluorophores into their structures. The highest emission peak is observed at a wavelength of 395 nm, with the excitation wavelength reaching its maximum at 350 nm. The blue emission has been observed with a maximum peak at 395 nm using an optimized excitation wavelength of 350 nm and represented with the help of a CIE plot in Figure S8.

### Solvent Effect on Emission Spectra

**Figure 3:**
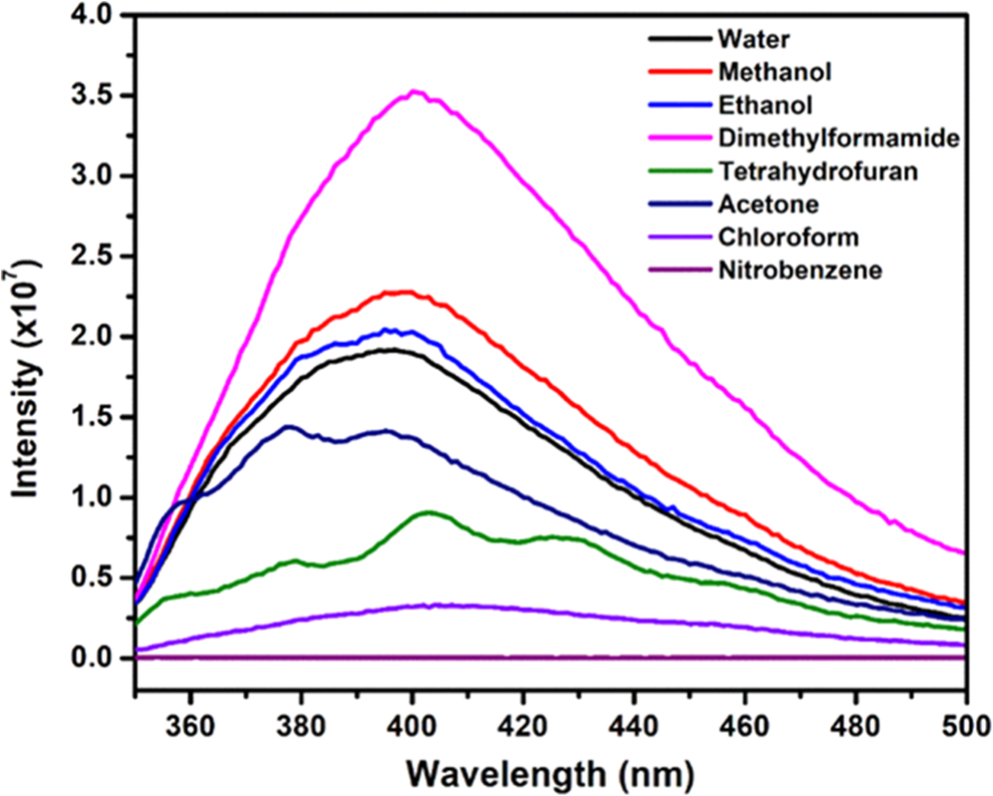
Normalized intensity of Cpz-4-HC in different solvents.

To comprehend the influence of solvents on the emission spectra of Cpz-4-HC, a range of solvents, including water, methanol, ethanol, DMF, THF, acetone, chloroform, THF, and nitrobenzene, were employed. Figure 3 displays the emission spectra of Cpz-4-HC when various solvents are present. Significantly, the emission intensity is greatest in DMF, subsequently followed by methanol. The order of fluorescence intensity is DMF > MeOH > EtOH > water > acetone > THF > CHCl_3_ > nitrobenzene. This variance in emission intensity and wavelength can be attributed to electron transfer processes and the interactions between the compound’s Cpz-4-HC framework and the respective solvents.^18^ It is worth mentioning that the emission intensity is entirely quenched in the presence of nitrobenzene. Despite the strong fluorescence response in DMF, the practical detection of TNP might require the use of water dispersion due to practical, environmental, and biocompatibility considerations.

### Fluorescence Sensing of TNP

**Figure 4:**
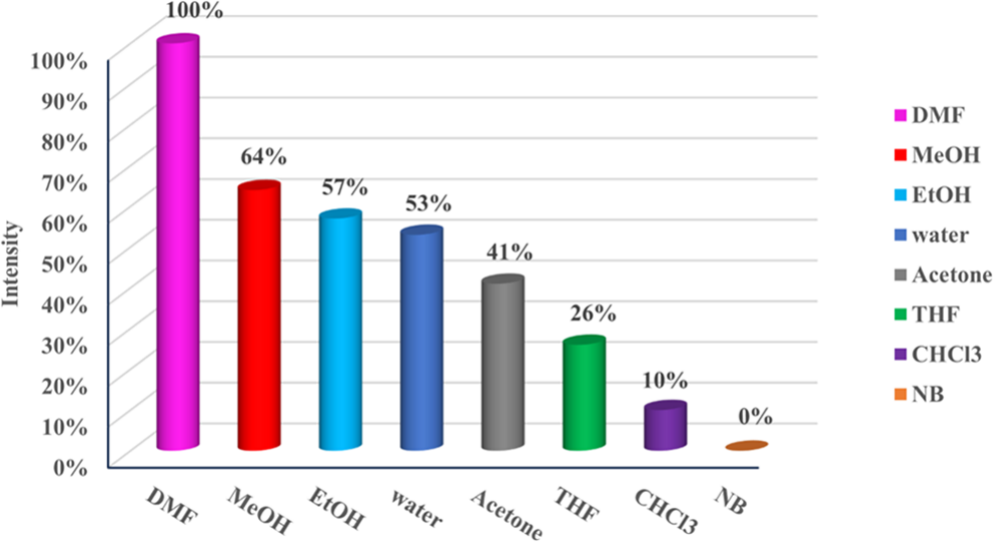
Fluorescence intensity ratio histograms of Cpz-4-HC dispersed in different solvents.

**Figure 5:**
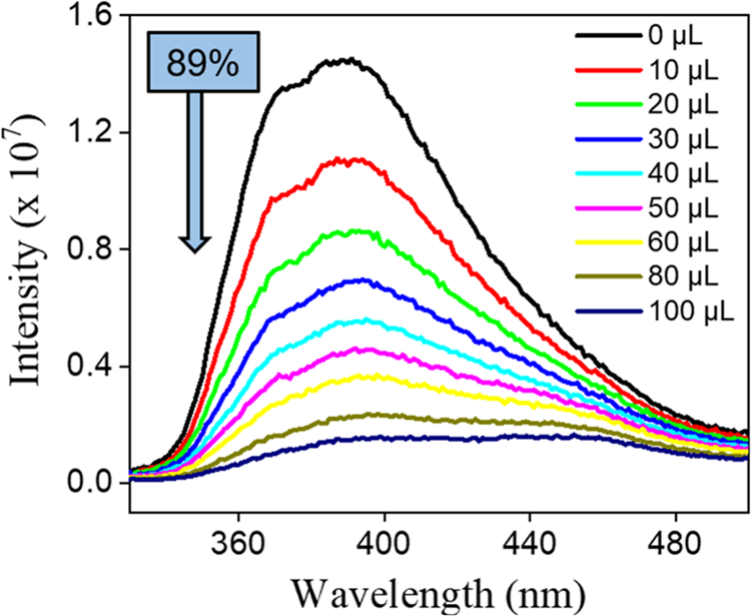
Emission spectrum of Cpz-4-HC dispersed in water after the addition of an aqueous 2,4,6-TNP solution.

To evaluate the ability to detect Cpz-4-HC in comparison to nitrophenols (NPs), we first examined the impact of nitrobenzene (NB) on the intensity of the emission of Cpz-4-HC in the specified solvents. Figure 4 clearly shows that NB quenches the emission intensity of Cpz-4-HC while remaining unaffected by the other solvents. Cpz-4-HC has the potential to be a useful approach for identifying NB and other nitrophenols in water. Fluorescence quenching titration experiments were conducted by gradually adding aqueous solutions (2 mM; 10–50 μL each) of various NACs to explore the sensing capabilities of Cpz-4-HC toward distinct NACs in water (Figure 4 and Section S-2; Figures S9–S14). The quenching efficiency percentage for different NPs was calculated using the formula
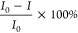
where *I*_0_ and *I* are the emission intensities of Cpz-4-HC before and after the incorporation of the NACs, respectively. Other NACs were shown to have a minor to slight quenching effect compared to TNP (Figure 5).

**Figure 6:**
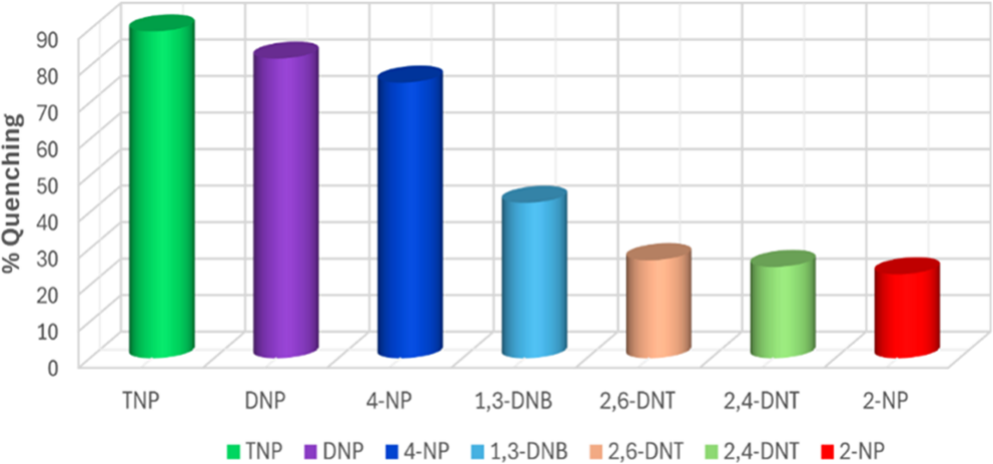
Percentage of fluorescence quenching obtained for different NACs at room temperature.

**Figure 7:**
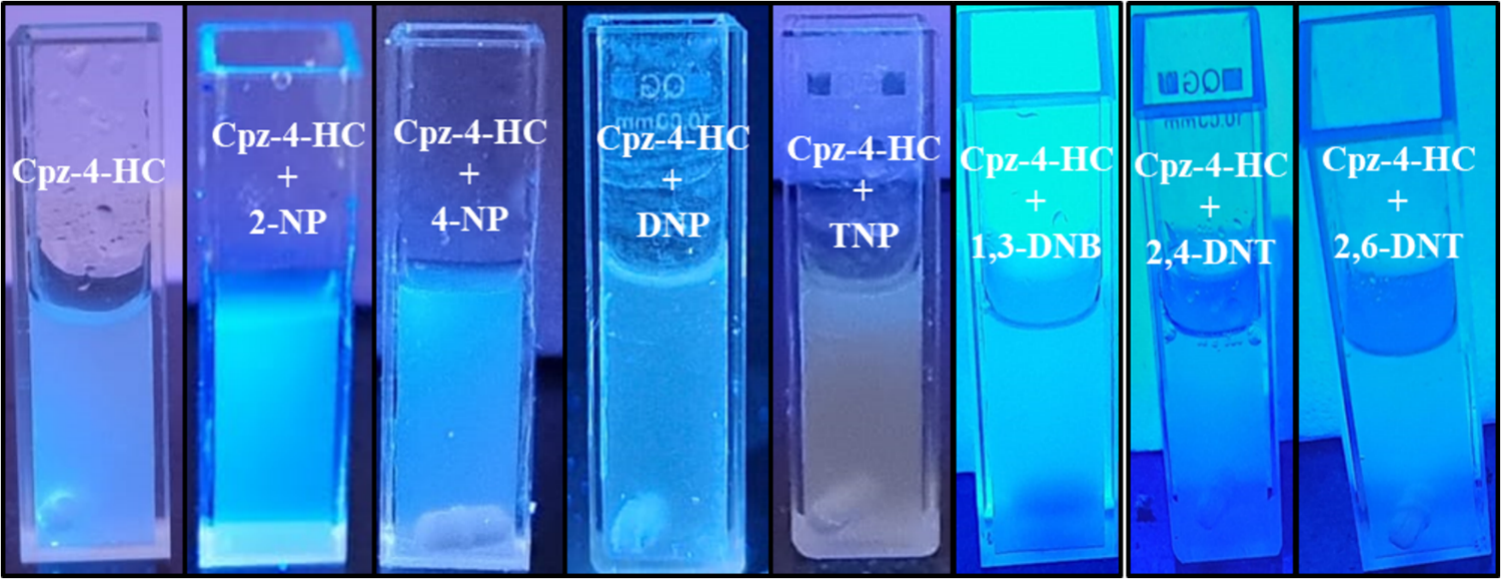
Response of Cpz-4-HC in the Presence of NACs by a UV-illuminator.

The order of fluorescence quenching efficiency of Cpz-4-HC is as follows: 2-NP < 2,4-DNT < 2,6-DNT < 1,3-DNB < 4-NP < 2,4-DNP < TNP, as shown in Figure 6. The quenching efficiency of Cpz-4-HC is further demonstrated through UV irradiation, enabling discrimination between TNP and 2,4-DNP, as shown in Figure 7. When TNP was added in small amounts (up to 50 μL) to finely powdered Cpz-4-HC, there was a remarkable and considerable reduction in fluorescence of approximately 90% (Figure 5). Surprisingly, the intensity of Cpz-4-HC dropped by 23.5% when 10 μL of TNP (2 mM stock solution) was added incrementally, as shown in Figure 4. The intensity dropped by approximately 75 and 90% when 60 and 100 μL of TNP (2 mM stock solution) were added, respectively.

**Figure 8:**
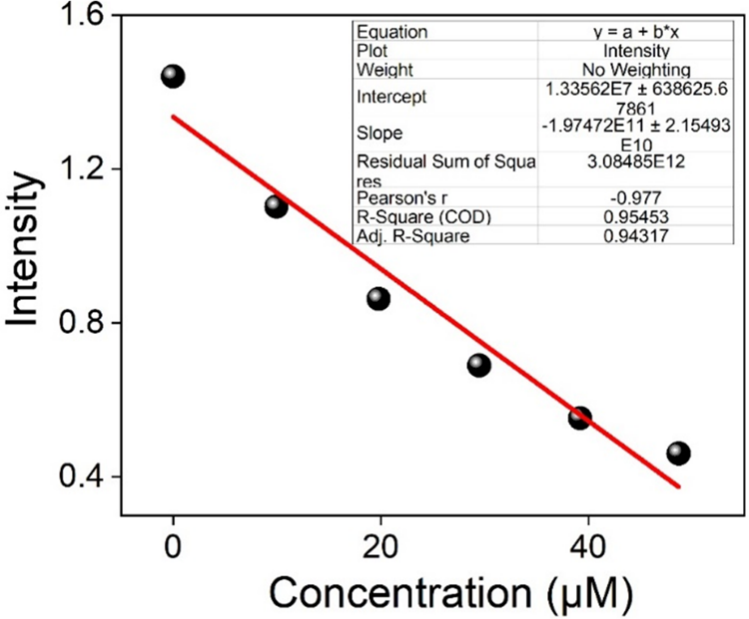
Linear region of fluorescence intensity of Cpz-4-HC upon incremental addition of TNP ((10–50 μL), 2 mM stock solution) at λ_em_ = 395 nm (upon λ_ex_ = 350 nm).

The minimum detectable concentration of TNP is determined to be as low as 0.334 ppm (ppm) based on the data shown in Figure 8 and Table S1. The findings clearly show that Cpz-4-HC prefers TNP over other possibly interfering NACs. The *S*–*V* plot of TNP for Cpz-4-HC demonstrates linearity at lower concentrations but deviates from linearity at higher values. This can be refined by employing the Stern–Volmer equation
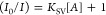
where

*I*_0_ = emission intensity in the absence of NPs,

*I* = emission intensity in the presence of NPs,

*K*_SV_ = quenching coefficient (M^–1^), and

[*A*] = molar concentration of the analyte, respectively.

**Figure 9:**
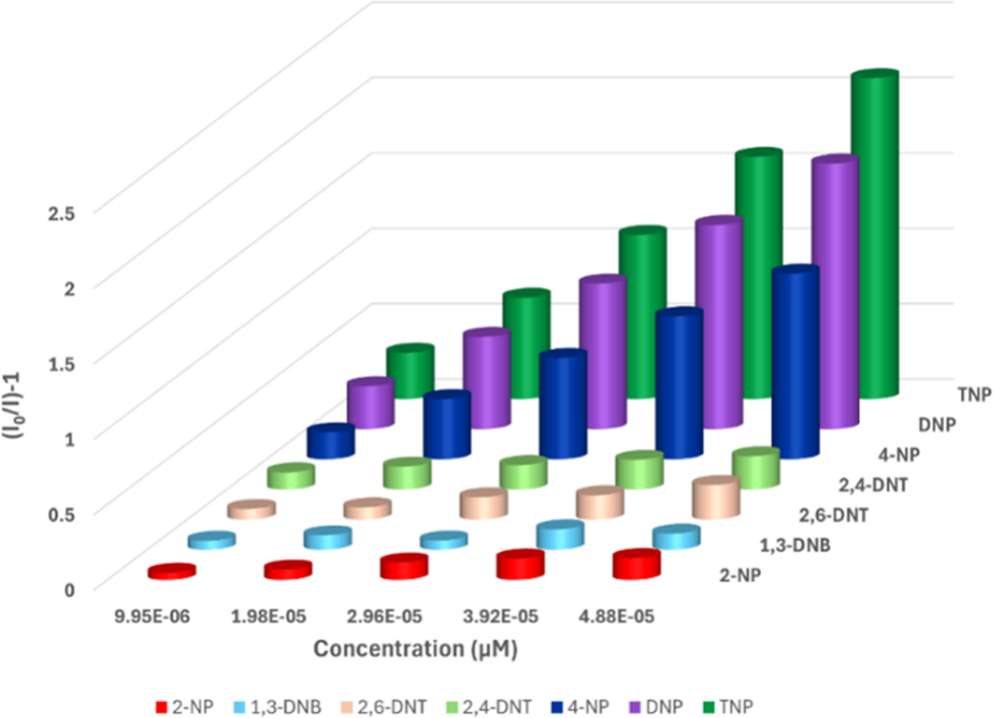
Stern–Volmer plot of Cpz-4-HC in the presence of TNP and other NACs.

In addition, the Stern–Volmer plots of the relative fluorescence intensity (*I*_0_/*I*) of Cpz-4-HC versus the analyte concentration are shown in Figure 9. Based on the slope of the linear Stern–Volmer plot of Cpz-4-HC, it is estimated to be 4.71 × 10^4^ M^–1^. Employing the formula (3σ/m), the detection limit of 2,4,6-trinitrophenol was found to be 0.334 ppm, which is comparable to other reported sensors for TNP so far (Table S2). Additionally, the limit of detection for Cpz-4-HC is found to be 0.334 ppm, which makes it highly desirable to detect TNP at low concentrations as compared to other probes found in the literature using water as a medium, which makes it environmentally friendly and effective for real-time applications compared to probes using a nonaqueous medium.

In the case of other NPs, a linear curve with an increase in concentration was observed in their respective SV plots (Section S4; Figures S15–S21, and Table S3). Cpz-4-HC strongly prefers TNP over other interfering NACs. The Stern–Volmer rate constant (K_sv_) for 2,4,6-TNP is 17 times higher than 2-nitrophenol, indicating better quenching ability of TNP toward luminescent Cpz-4-HC in water. As a result, Cpz-4-HC is an ideal choice for environmental monitoring (Table S2).

**Figure 10:**
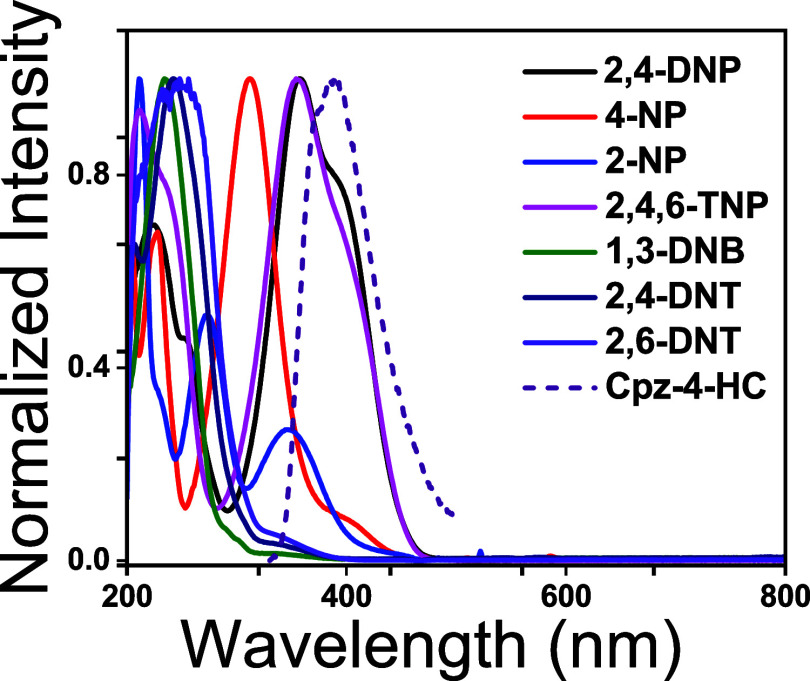
Spectral overlap between the absorption spectra of various nitrophenols and the emission spectra of Cpz-4-HC.

The spectral overlap between the absorbance spectra of the NACs (2 mM) and the emission spectra of Cpz-4-HC was analyzed to further understand the sensing mechanism, as depicted in Figure 10. Greater quenching efficiencies of NACs were indicated by a larger extent of overlap. Overlap was noted more significantly in the case of TNP, followed by that of 2,4-DNP, whereas no overlap was observed for other NACs. This confirms the resonance energy transfer, a long-range phenomenon, from the electron-rich sensor Cpz-4-HC to the electron-deficient acidic nitrophenol derivatives, leading to a significant subsequent increase in the quenching efficiency.

Fluorescence lifetime study experiments were finally conducted to differentiate between the two potential quenching processes (static and dynamic).^19^ Typically, the decrease in the fluorescence lifetime of the chemosensor is attributed to a dynamic quenching process caused by diffusion-mediated collisions between the excited sensor and the quencher, while a constant fluorescence lifetime indicates static quenching. In static quenching, the fluorescent molecules create a nonfluorescent complex, or “dark state”, with the quenching agent, leaving the unconnected fluorescent molecules to display their natural lifetime.

**Figure 11:**
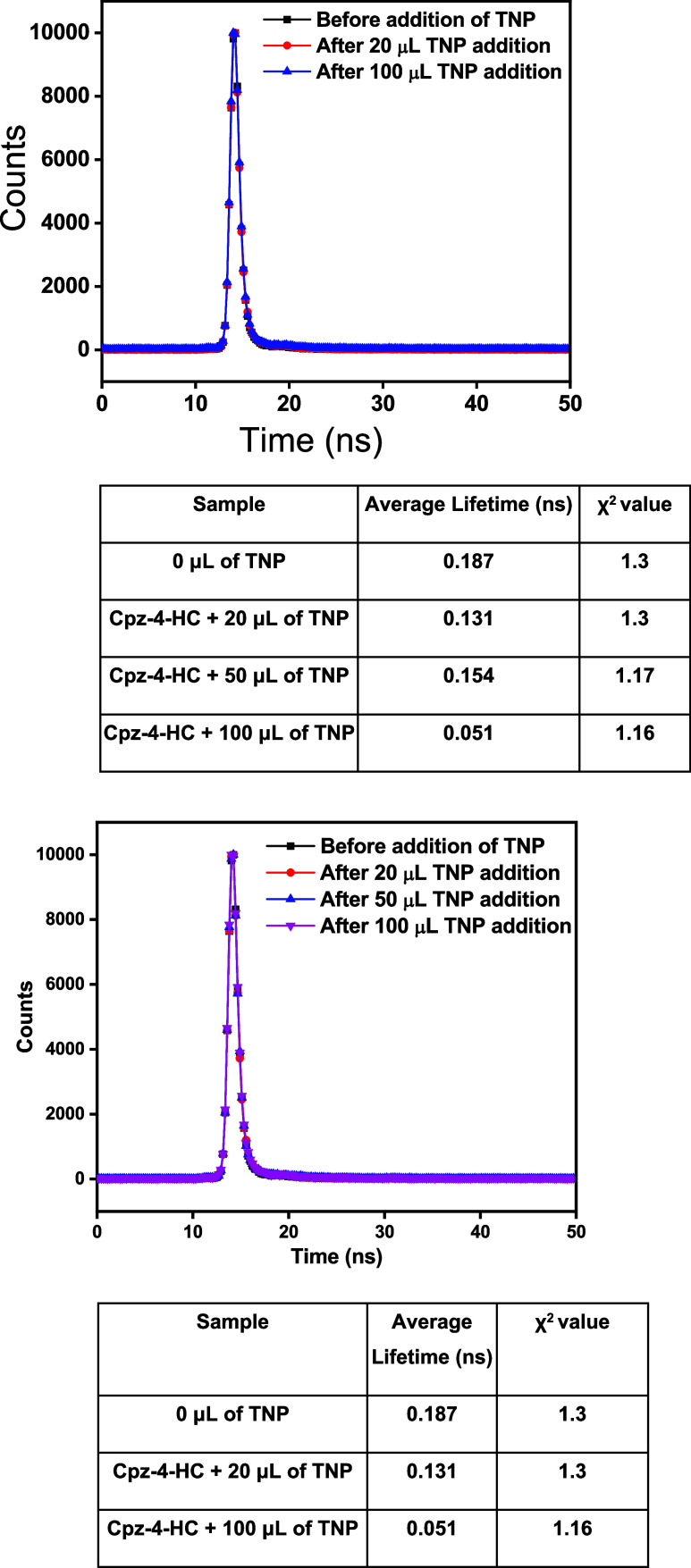
Fluorescence lifetime decay profiles of Cpz-4-HC before and after the incremental addition of TNP.

Time-resolved fluorescence experiments were conducted (Figure 11) to further clarify the quenching mechanism of the TNP based on the concentration-dependent average lifetimes of each probe. Lifetime decay studies were carried out by incrementally adding TNP to Cpz-4-HC. From the decay experiments, it was noted that the average lifetime (τ) values decreased from 0.187 to 0.051 ns upon the addition of 100 μL of TNP.

The quenching process was further clarified using time-resolved fluorescence experiments, which involved measuring the average lifetime of Cpz-4-HC’s excited state before and after introducing various concentrations of TNP. The average lifetimes are listed as follows: 0.187 ns (before the addition of TNP), 0.131 ns (after the addition of 20 μL of TNP), 0.154 ns (after 50 μL addition of TNP), and 0.051 ns (after 100 μL addition of TNP). The significant decrease in the lifetime value indicates that Cpz-4-HC is predominantly subjected to dynamic quenching upon the addition of TNP. The fluorescence lifetime of the system remained nearly unchanged from 0.131 to 0.051 ns after the addition of TNP, which met the phenomenon of the inner-filter effect (IFE). These observations suggest that the TNP-induced fluorescence quenching mechanism can be attributed to IFE.^20^

In the process of sensing, organic–inorganic hybrid sensors utilize various intricate detection mechanisms, including but not limited to photoinduced electron transfer (PET), resonance energy transfer, competitive absorption (CA), structural transition (ST), and chemical transformation.^21^

**Figure 12:**
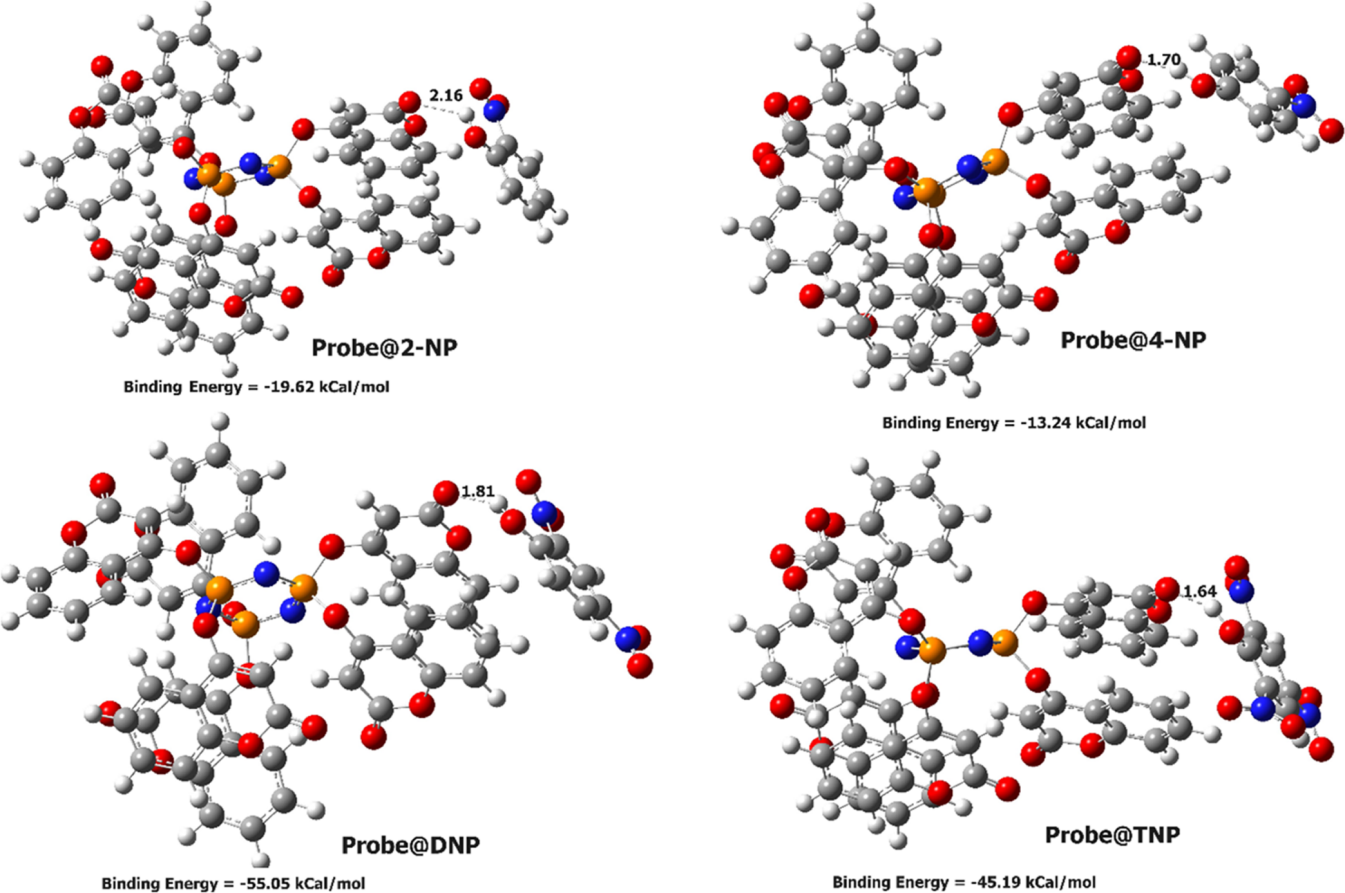
Optimized geometry of the complexes with the binding energy and the shortest distance (in Å) between the probe and the molecule.

**Figure 13:**
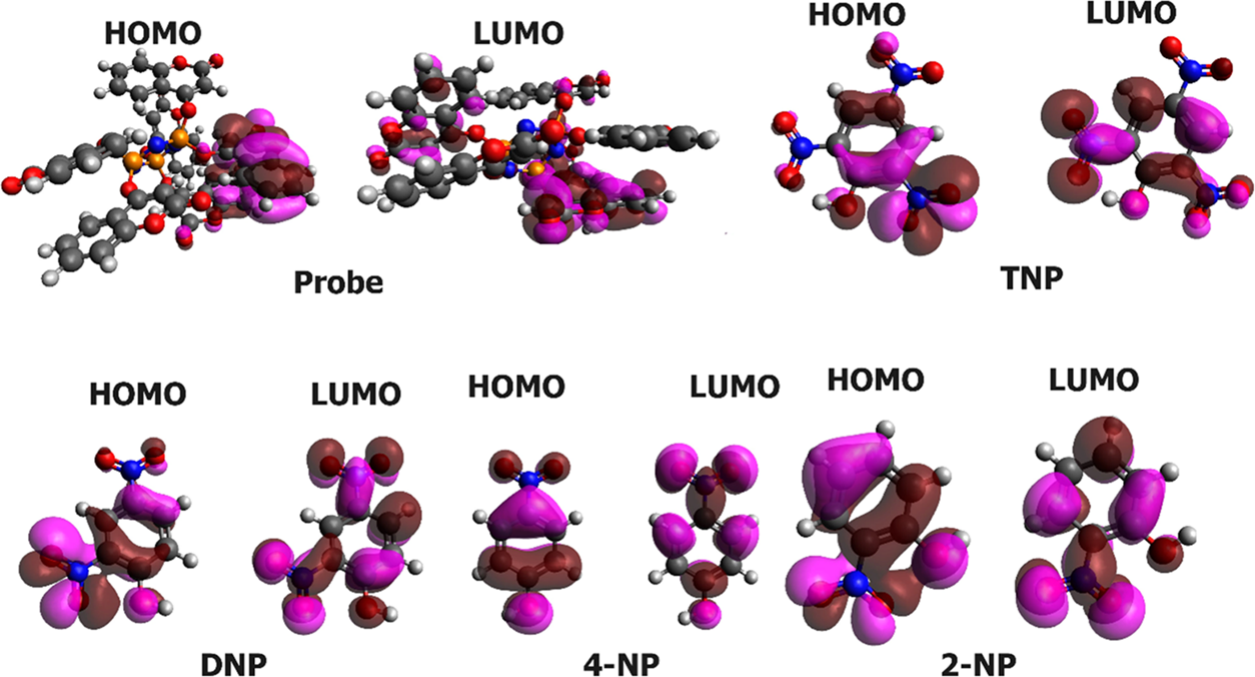
Spatial distribution of the molecular orbitals of isolated molecules.

The optimized geometries of the complexes are shown in Figure 12, along with their binding energies and the shortest distances between the –C=O of the probe and the −OH group of the molecules. It has been found that TNP has the shortest distance of 1.64 Å, suggesting possible strong H-bonding compared to that of the other molecules. The DNP molecule exhibits the highest binding energy of −55.05 kcal/mol, whereas, for TNP, it is −45.19 kcal/mol. This is attributed to steric interactions of the two –NO_2_ groups in the ortho position in TNP. The binding energy is dependent on the highest occupied molecular orbital–lowest unoccupied molecular orbital (HOMO–LUMO) gap of complexes.^22^ A higher HOMO–LUMO gap gives higher binding energy, and a lower HOMO–LUMO gap gives lower binding energy. If we see the figure of the HOMO–LUMO gap, we can see that the HOMO–LUMO gap of the TNP complex is lower than the DNP complex, which explains the higher binding energy of the DNP complex than the TNP complex. Figure 13 displays the HOMO and LUMO of the isolated probe and the molecules.

**Figure 14:**
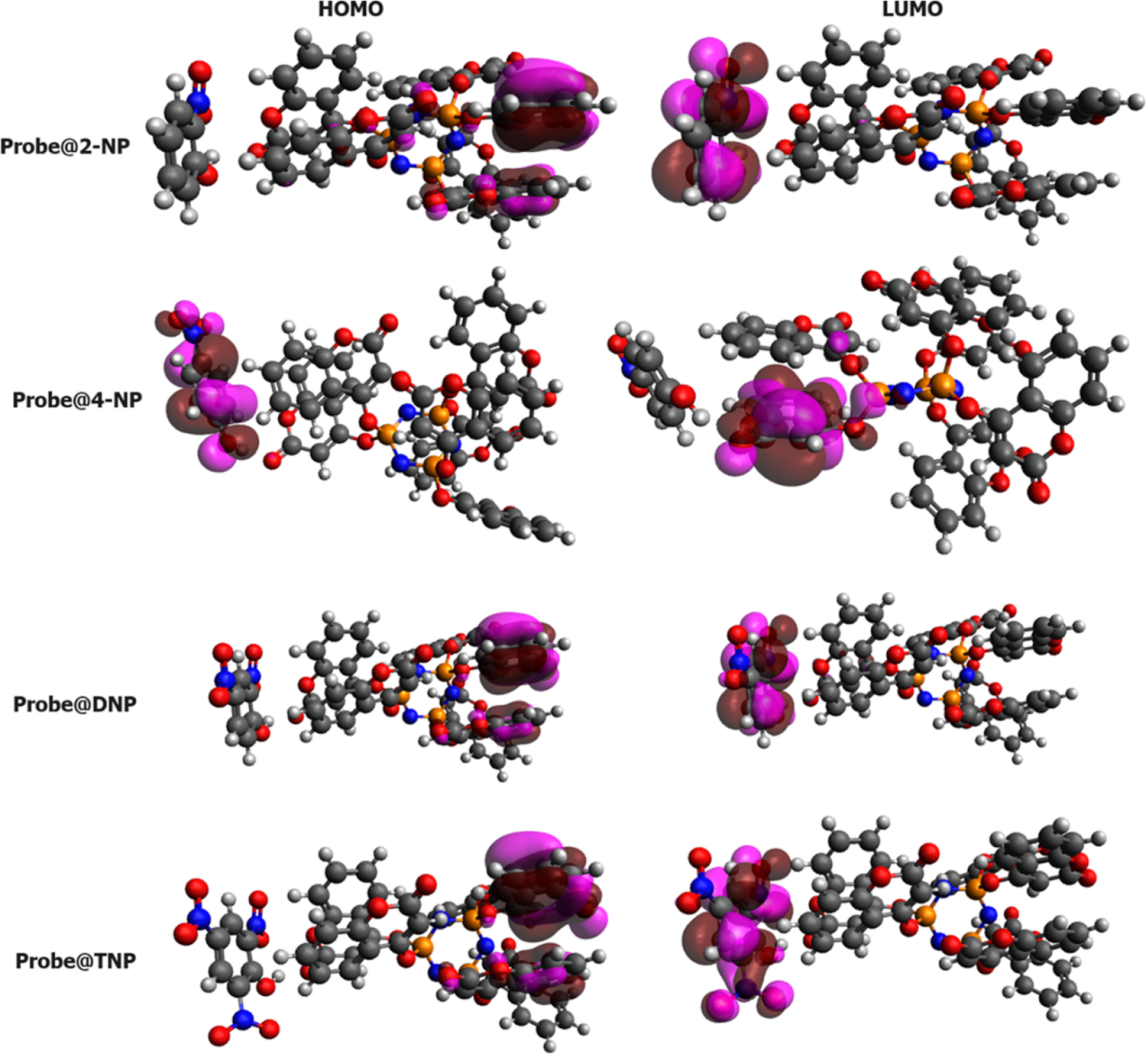
Spatial distribution of molecular orbitals of the complexes.

Experimental values of the photophysical parameters are provided in Table 1, indicating that TNP exhibits the highest sensitivity toward detection, as evidenced by the quantum efficiency and detection limit. Figure 14 illustrates the HOMO and LUMO of the probe@molecule (where probe = Cpz-4-HC whereas molecule = TNP, DNP, 4-NP, and 2-NP, respectively). It is observed that the HOMO is on the probe, and the LUMO is on the molecule, except for the probe@4-NP, in which case the opposite is true.

**Table 1:** Experimental Photophysical Data for the Probes

probe	*l*_abs_(nm)	*l*_emi_(nm)	quantum efficiency (%)	detection limit (PPM)
probe@2NP	210	395	23	0.969
probe@4NP	310	395	75.47	0.476
probe@DNP	370	395	82	0.757
probe@TNP	370	395	89.49	0.334

**Figure 15:**
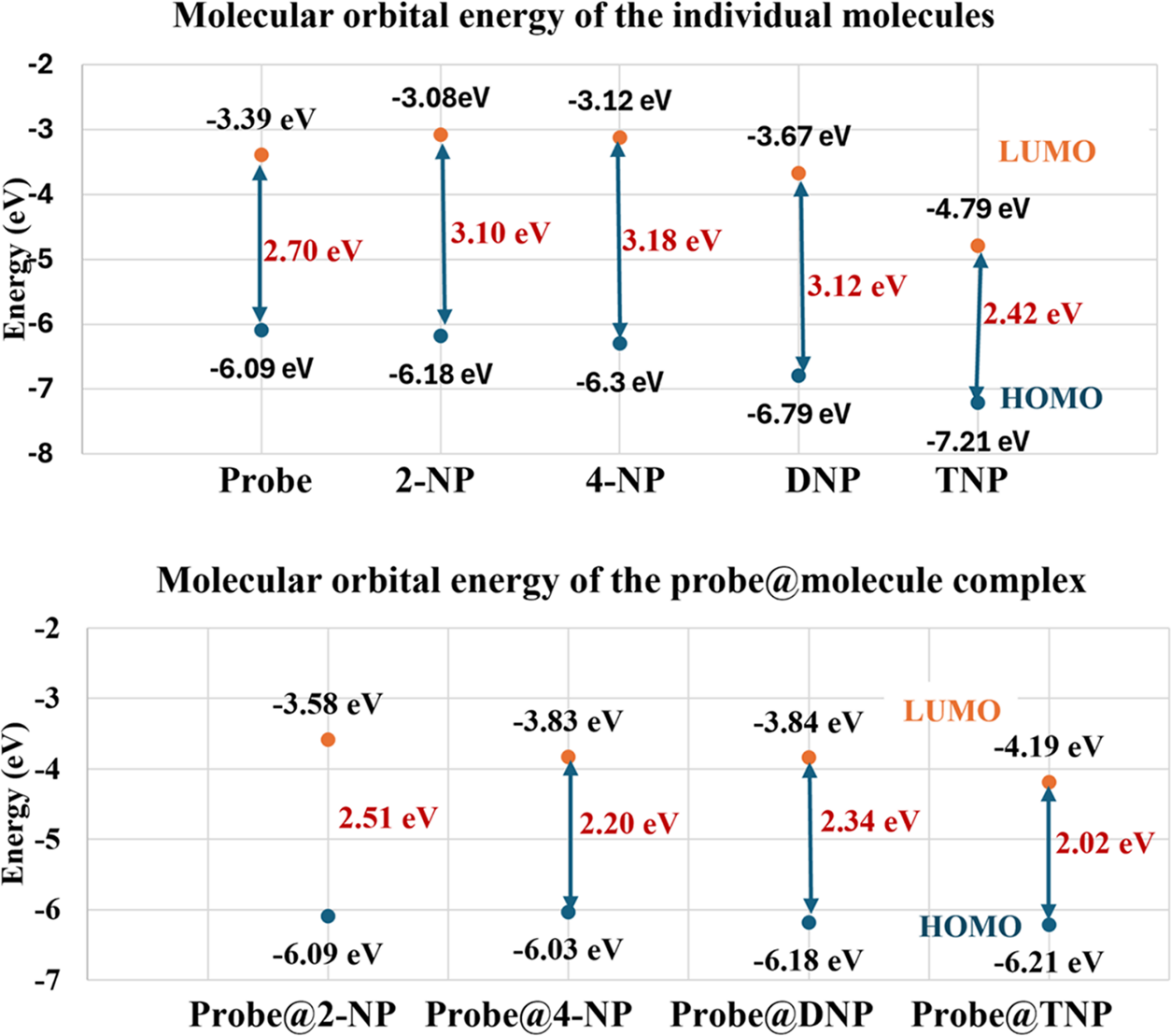
Molecular orbital energy diagram of the isolated molecules and the probe@molecule complexes.

The photochemical phenomenon occurs due to an electron transition from the HOMO to the LUMO, depending on the HOMO–LUMO energy gap. The energy gaps of the probe, isolated molecules, and probe@molecules are presented in Figure 15. From Figure 15, it can be seen that the HOMO of the probe is closer to the LUMO of TNP compared to other molecules, indicating that electron transition from the HOMO of the probe to the LUMO of TNP is much easier, as supported by the experimental findings. The LUMO energies of 2- NP and 4-NP were higher than that of the probe Cpz-4-HC, indicating a mismatch in energy levels between the analytes and the probe. Consequently, the quenching effect was not likely to occur via an electron transfer. The LUMO of an electron-rich probe Cpz-4-HC lies at a higher energy level (−3.39 eV) compared to that of the electron-deficient analyte TNP (−4.79 eV), as shown in Table 2. So, there is a high probability of electron transfer from the LUMO containing excited electrons of the probe Cpz-4-HC to the LUMO of the analyte TNP, leading to fluorescence quenching. This observation aligns with the principles of the PET mechanism.

**Table 2:** Calculation Data of the LUMO and HOMO Energies of Probe Cpz-4-HC and Nitro-phenolic Organic Small Molecules

analytes	HOMO (eV)	LUMO (eV)
probe Cpz-4-HC	–6.09	–3.39
2-NP	–6.18	–3.08
4-NP	–6.3	–3.12
DNP	–6.79	–3.67
TNP	–7.21	–4.79

**Figure 16:**
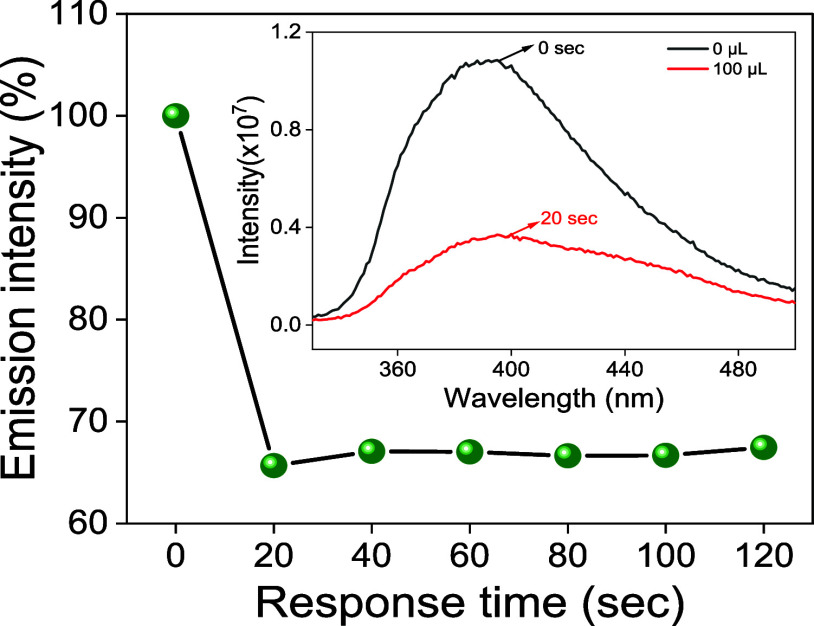
Percentage of emission intensity at different response times (s) for Cpz-4-HC; inset: emission spectra of Cpz-4-HC before (0 s) and after (20 s) addition of 50 μL of TNP; λ_exc_ = 330 nm.

For effective real-time sensing, it is essential for a sensor to quickly detect the analyte. We demonstrated that the Cpz-4-HC probe enabled ultrafast detection of TNP, with a substantial decrease in emission intensity occurring within just 20 s after adding 50 μL of TNP to its homogeneous dispersion, as shown in Figure 16. Additionally, the Cpz-4-HC probe’s ultrafast detection of TNP was confirmed by the consistent emission intensity observed at a 20 s interval up to 120 s.

**Figure 17:**
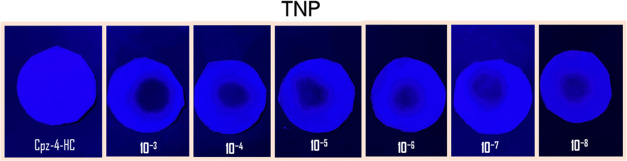
Test paper strips for the detection of TNP under 365 nm UV light.

Test strips made from Whatman filter paper were soaked in aqueous dispersions of Cpz-4-HC and then air-dried. Under UV light (λ = 365 nm), these test strips appeared dark blue. When varying concentrations of TNP (10^–3^ – 10^–8^ M) were drop-cast onto the test strips, the blue color darkened, indicating a quenching of the fluorescence intensity, as shown in Figure 17.

**Figure 18:**
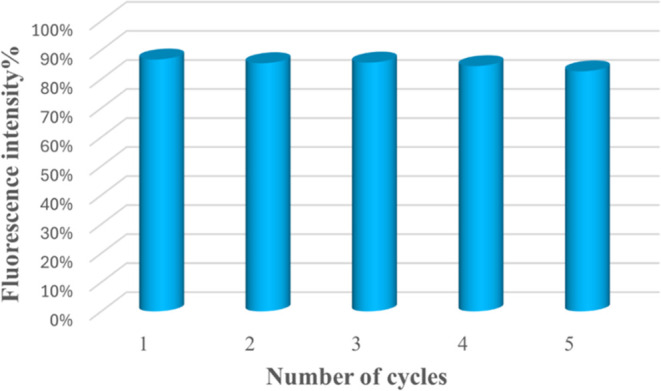
Recyclability test for Cpz-4-HC in the presence of TNP.

The recyclability experiment of probe Cpz-4HC with TNP was conducted for up to five cycles. In order to investigate the recyclability, the dispersion was centrifuged, filtered, and washed several times with water and methanol to prepare for the next cycle. The results showed that for up to five repeated cycles, the probe with TNP regained its fluorescence intensity and quenching efficiency without any significant change (Figure 18).

**Figure 19:**
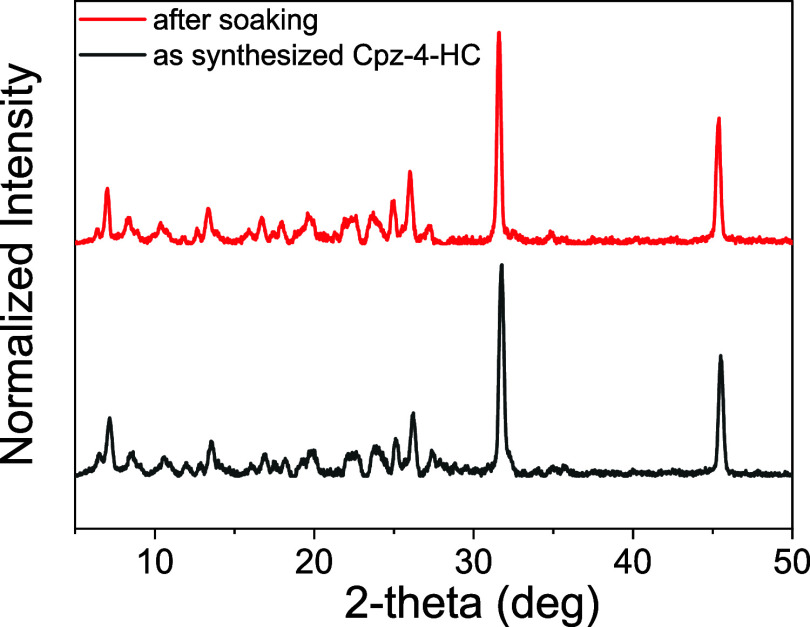
PXRD patterns of Cpz-4-HC before and after soaking in TNP for 3 days.

The PXRD patterns recorded before and after immersing Cpz-4-HC in an aqueous solution of TNP for 3 days showed no significant change (Figure 19). This confirms that the structure of Cpz-4-HC remains unaltered during the sensing experiments.

## Conclusions

In conclusion, this study synthesized and characterized 4-hydroxycoumarin-substituted cyclotriphosphazene (Cpz-4-HC) through a single displacement reaction. The compound was purified using column chromatography and preparative TLC, and its chemical structure was analyzed using various techniques, including HRMS mass spectrometry, FT-IR spectroscopy, and NMR spectroscopy. The FT-IR spectrum confirmed the presence of both HCCP (**1**) and 4-hydroxycoumarin (**2**) in Cpz-4-HC, while the NMR spectra indicated structural modification. The compound exhibited photophysical properties with absorption and emission peaks, and different solvents influenced its emission intensity. In spectrofluorometric titrations, Cpz-4-HC was found to have an efficient, sensitive, and highly selective fluorescence quenching response to TNP compared with some of the other interferents, including 2,4-DNP, 4-NP, and 2-NP. Furthermore, Cpz-4-HC showed high selectivity for the detection of nitroaromatic compounds (NEs) in water, with a significant fluorescence quenching effect observed for TNP. The detection limit for TNP is found to be 0.334 ppm, emphasizing its sensitivity, and the *S*–*V* plot demonstrates linearity at lower concentrations. The *S*–*V* plot further confirms this selectivity, with a *K*_SV_ value of 4.71× 10^4^ M^–1^ obtained for Cpz-4-HC. Therefore, Cpz-4-HC has the potential for the detection of TNP and other nitrophenols. The quenching mechanism, elucidated through fluorescence lifetime studies, suggests a dynamic quenching process that is primarily attributed to electron transfer.

Drop-casting various concentrations of TNP solutions onto test paper strips soaked in an aqueous dispersion of **Cpz-4-HC** resulted in a noticeable change in color intensity. Cpz-4-HC was recyclable for up to five cycles without much loss in sensitivity. Additionally, computational simulations support the interaction between Cpz-4-HC and TNP, indicating possible hydrogen bonding and favorable energy level alignment for photoinduced electron transfer. Overall, Cpz-4-HC presents itself as a promising candidate for the selective and sensitive detection of TNP, offering insights into potential applications in environmental sensing and monitoring efforts.

## Experimental Section

### Materials and Methods

Hexachlorocyclotriphosphazene, 4-hydroxycoumarin, potassium carbonate (K_2_CO_3_), and sodium hydride (NaH) were purchased from Sigma-Aldrich. Tetrahydrofuran (THF) and dichloromethane (DCM) were procured from Merck. Silica gel (100–200) was employed for column chromatographic separations, while alumina sheets precoated with silica gel (Merck, Kieselgel 60, F254) were used for thin-layer chromatography (TLC). The Buchi M-565 Melting Point Apparatus was used to determine the melting point.

**Caution! TNP is highly explosive and should be handled with caution in small amounts**. To avoid an explosion, handling explosives requires precautions and should be done in dilute solutions.

### Measurement and Characterization

#### Structural Characterization

^1^H NMR, ^31^P NMR (400 MHz), and ^13^C NMR (101 MHz) spectra in DMSO-*d*_6_ were recorded at room temperature using a Bruker Biospin Switzerland Avance-iii 400 MHz spectrometer. Tetramethylsilane (TMS) was used as an internal standard for ^1^H- and ^13^C NMR. In the case of ^31^P NMR, δ values were recorded relative to external 85% aqueous H_3_PO_4_.

#### Fourier-Transform Infrared (FT-IR) Spectroscopy

FT-IR spectra of the compounds by the ATR technique were recorded on a Shimadzu FT-IR spectrometer in the range of 400 to 4000 cm^–1^.

#### UV–vis Measurements

UV–vis-NIR and solid-state reflectance spectra were recorded using the Cary 5000 spectrophotometer by Agilent Technology for UV–vis spectra measurements.

#### Powder X-ray Diffraction (PXRD)

Powder X-ray diffraction (PXRD) measurements were done on a Rigaku Miniflex 600 (5th gen) X-ray diffractometer with Cu Kα X-ray radiation.

#### Scanning Electron Microscope (SEM)

The SEM images were recorded using a ZEISS EVO MA18 at an operating voltage of 10 kV.

#### Thermogravimetric Analysis (TGA)

Thermogravimetric analysis (TGA) was carried out on a Shimadzu DTG-60 instrument under a nitrogen atmosphere with a heating rate of 10 °C min^–1^ over a temperature range of 25 to 600 °C by using an aluminum pan.

#### Fluorescence Spectroscopy

Fluorescence measurements were performed by using a HitachiF7000 spectrophotometer (Luma 40) from Quantum Northwest. The fluorescence spectra were measured using a Horiba Scientific Fluorolog 3 Spectrophotometer. The lifetime measurement of Cpz-4-HC was measured using a time-resolved HORIBA scientific single photon counting controller.

### Synthesis of Cpz-4-HC

The facile, straightforward preparation of Cpz-4-HC was carried out by a one-step single displacement method, as shown in Scheme 1. A suspension of sodium phenolate suspension was prepared by adding sodium hydride (287.63 mg, 10 equiv) to 4-hydroxycoumarin (932.75 mg, 8 equiv) dissolved in 40 mL of dry THF under an argon environment. Hexachlorocyclotriphosphazene (250 mg, 1 equiv) was then added dropwise with a syringe to 10 mL of dry THF, and the resulting mixture was agitated under reflux conditions for 2 days. Following solvent removal at decreased pressure, the residual component was purified using column chromatography with silica gel and an eluent composed of hexane and ethyl acetate (v/v 100/80). The yield is 65%, and the melting point is 292.9 °C.

HRMS (ESI) *m*/*z*: calcd for C_54_H_30_N_3_O_18_P_3_ 1102.0914; found 1124.0914 [M + Na]^+^. FT-IR (cm^–1^): υ(C=O) = 1728 cm^–1^, (υ(C–C) _arom_) = 1625 cm^–1^, (ν(C–H) _arom_) 3371, υ(P=N) = 1222 cm^–1^, υ(P–N) = 772 cm^–1^, υ(P–O) = 1079 cm^–1^, *ν(*C–O–C) 1127 cm^–1^.^1^H NMR (400 MHz, DMSO-*d*_6_) δ 7.64 (t, *J* = 8.5 Hz, 6H_d_), 7.54 (d, *J* = 7.9 Hz, 6H_b_), 7.36 (d, *J* = 8.2 Hz,6H_e_), 7.21 (t, *J* = 7.6 Hz,6H_c_), 6.53 (s, 6H_a_).^13^C NMR (101 MHz, DMSO) δ 173.75, 160.60, 153.35, 133.95, 124.25, 123.43, 117.07, 116.78,106.92. ^31^P NMR (500 MHz, CD_2_Cl_2_) δ, ppm: 5.64.

### Fluorescence Study

In a standard solvent analysis experiment, 1 mg of finely ground Cpz-4-HC was mixed with 2 mL of a specified solvent contained in a cuvette of quartz with a pathway diameter of 10 mm. The mixture was continuously agitated to create a uniformly dispersed suspension, which was then used to measure the emission spectrum. In the fluorescence titration experiment, seven different nitroaromatics, specifically 2,4,6-trinitrophenol (TNP), 2,4-dinitrophenol (2,4-DNP), 4-nitrophenol (4-NP), 2-nitrophenol (2-NP), 2,6-dinitrotoluene (2,6-DNT), 2,4-dinitrotoluene (2,4-DNT), and 1,3-dinitrobenzene (1,3-DNB), were utilized as analytes. In each titration experiment, 1 mg of Cpz-4-HC was dispersed in 2 mL of Milli-Q water in a quartz cuvette. When Cpz-4-HC was excited at 350 nm, its fluorescence response was observed in the 330–800 nm range. Upon stirring for 10 min to form a uniformly dispersed slurry of Cpz-4-HC, each analyte solution was then added incrementally from a 2 mM stock solution, which was prepared in 9 mL of Milli-Q water and 1 mL of methanol to measure the resulting emission spectra. The emission intensity at 395 nm for Cpz-4-HC was measured. To ensure a uniformly distributed solution of Cpz-4-HC, the experiment maintained a consistent stirring rate. The experiments were conducted three times, yielding consistent outcomes.

### Detection Limit Calculation

To determine the detection limits, the fluorescence intensity was measured after exposure of Cpz-4-HC to the various nitrophenol-based analytes (2 mM stock solution). The slope (m) was calculated by plotting the fluorescence intensity against increasing concentrations of nitrophenols. The standard deviation (σ) of Cpz-4-HC was determined by using four blank measurements. Employing the formula (3σ/m), the detection limit was then calculated.

### Computational Details

All molecular geometries have been optimized using Orca 5.0 software.^17^ The calculations utilized the BP86 functional in combination with the def2-SVP basis set and the D3BJ dispersion correction. Avogadro software is used for the visualization of molecular orbitals.^18^

### Preparation of Test Strips

For rapid and on-site detection of TNP, test strips were prepared by using commercial Whatman filter paper. When soaked in aqueous dispersions of Cpz-4-HC, the strips exhibited a blue color under UV light. These coated strips were then employed for real-time detection of TNP by drop-casting a small volume of various TNP concentrations onto the strips.

## Supporting Information Available

Details of characterization of Cpz-4-HC: FT-IR spectra, ^1^H NMR, ^13^C NMR and ^31^ P NMR, HRMS spectrum, solid-state absorption spectrum, and fluorescence spectra and CIE 1931 plot (Section S-1); details of selective detection of TNP by Cpz-4-HC (Section S-2); details of calculation of detection of limit (Section S-3); details of Stern–Volmer plot of Cpz-4-HC (Section S-4); comparison of Cpz-4-HC with other reported TNP sensor (Table S2); and summary of Stern–Volmer rate constant (*K*_sv_) of Cpz-4-HC for different NACs at room temperature (Table S3) (PDF)
