# Supplementary material for: Novel Coumarin-Substituted Cyclophosphazene as a Fluorescent Probe for Highly Selective Detection of 2,4,6-Trinitrophenol
Source: ACS Omega. 2025 Feb 3;10(6):5312–23. doi: 10.1021/acsomega.4c05306 (PMC11840767; doi:10.1021/acsomega.4c05306)
Supplement: Supplementary file 1 — ao4c05306_si_001.pdf [file ao4c05306_si_001.pdf]

Supporting Information

"Novel Coumarin-substituted Cyclophosphazene as a fluorescent probe for highly selective Detection of 2,4,6-Trinitrophenol".

Ishanki Sharma[a], Rajeev Kumar Sinha[b], Suranjan Shil[c], Shruti Rani[d] and N.V. Anil Kumar*[a]

[a] Department of Chemistry, Manipal Institute of Technology, Manipal Academy of Higher Education, Manipal - 576104, India.

[b] Department of Physics, Birla Institute of Technology Mesra, Ranchi- 835215, India

[c] Manipal Centre for Natural Sciences, Manipal Academy of Higher Education, Manipal - 576104, India.

[d] Department of Chemical Sciences, Indian Institute of Science, Education and Research (IISER) Mohali

*nv.anil@manipal.edu

Section S-1: Characterization Of Cpz-4-HC

1. FT-IR

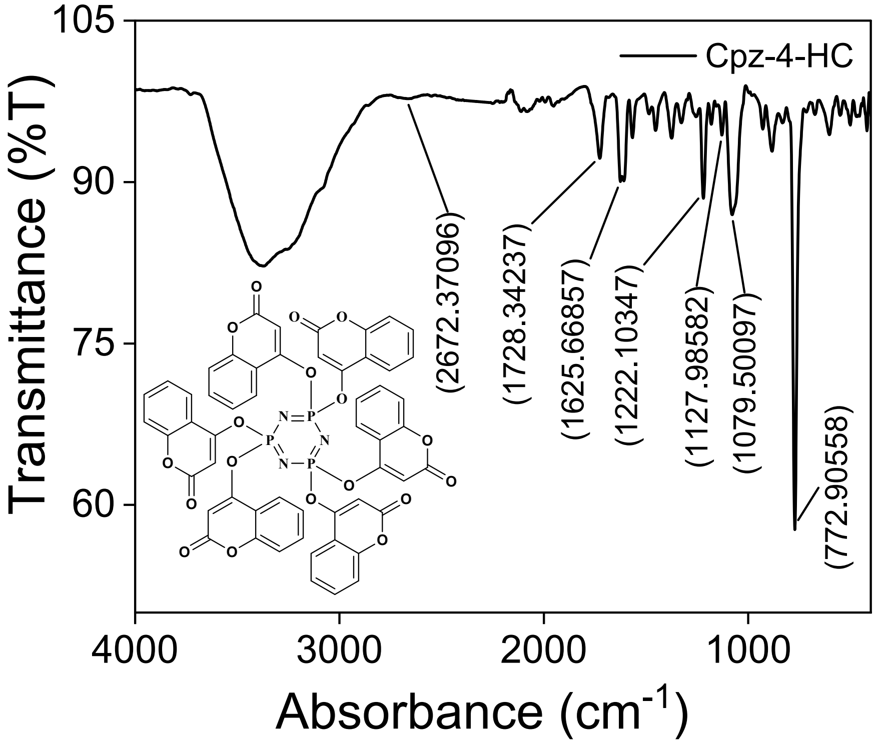

Fig. S1. FT-IR Spectra of **Cpz-4-HC**

2. N.M.R Spectra

2.1. ^{1}H N.M.R

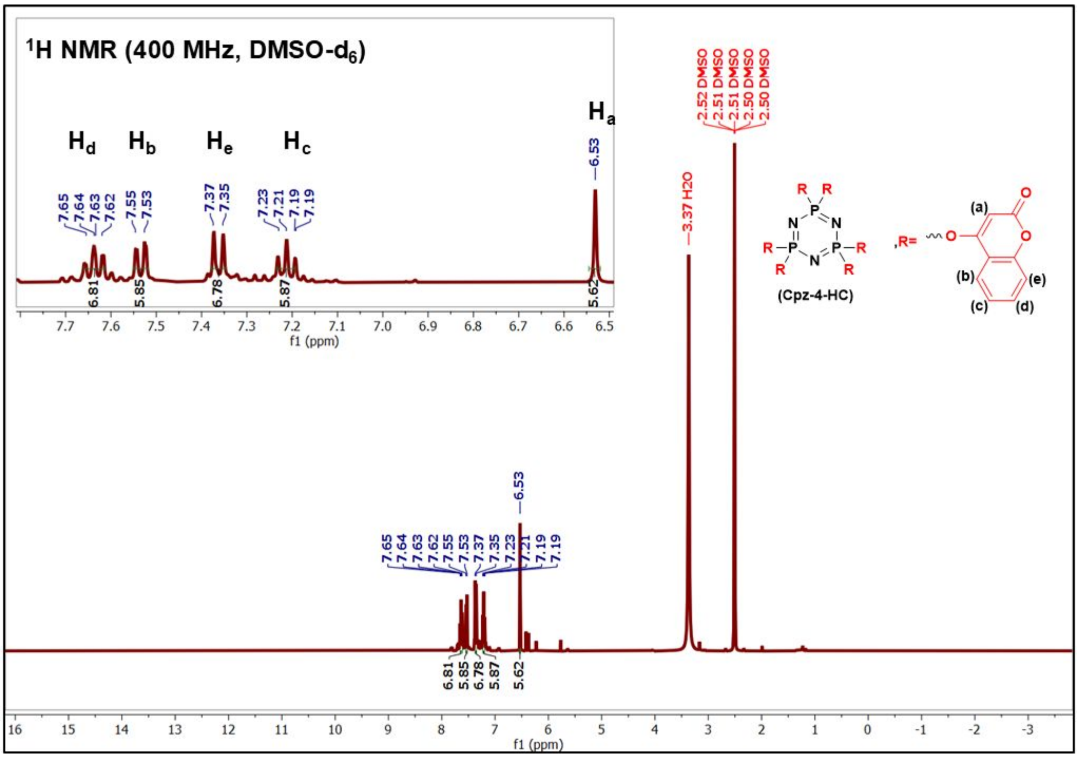

Fig. S2. ^{1}H N.M.R of **Cpz-4-HC**

2.2.^{13}C N.M.R.

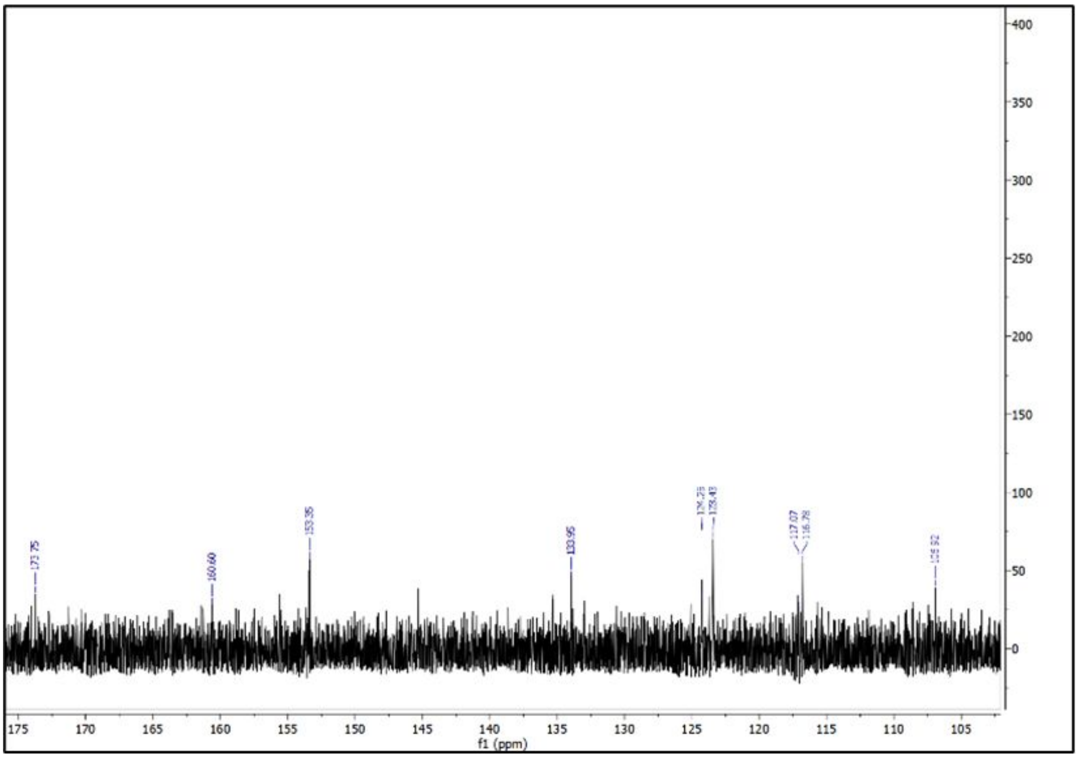

Fig. S3. ^{13}C N.M.R of **Cpz-4-HC**

2.3. ^{31}P N.M.R.

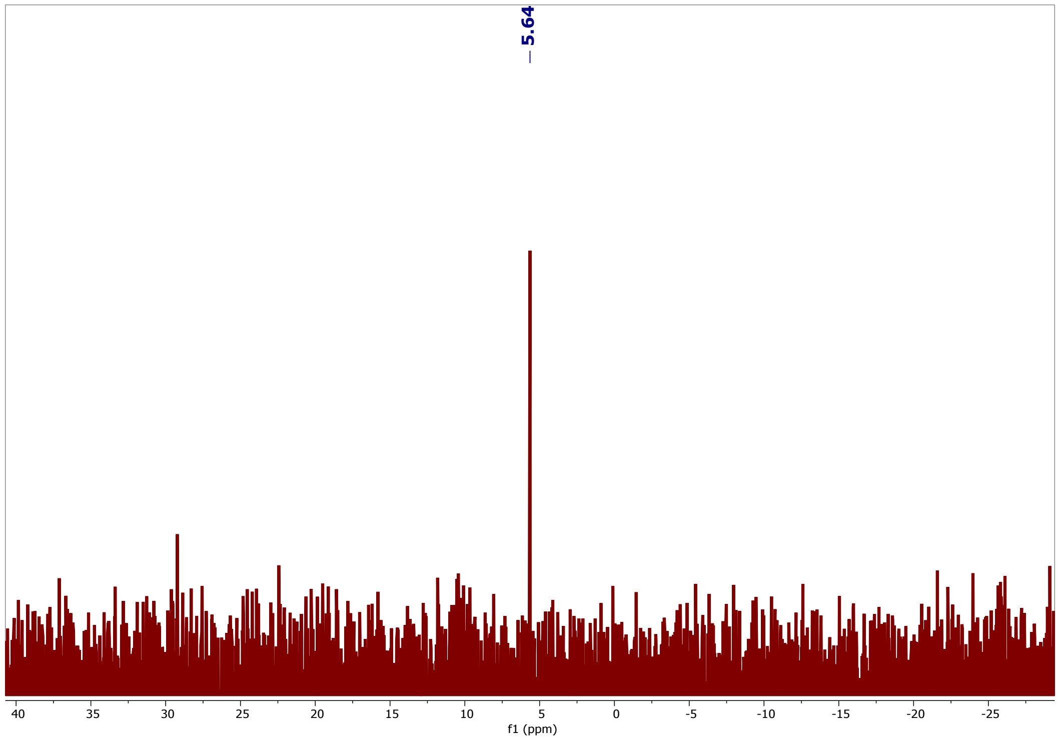

Fig. S4. ^{31}P N.M.R.

3. Mass Spectrum

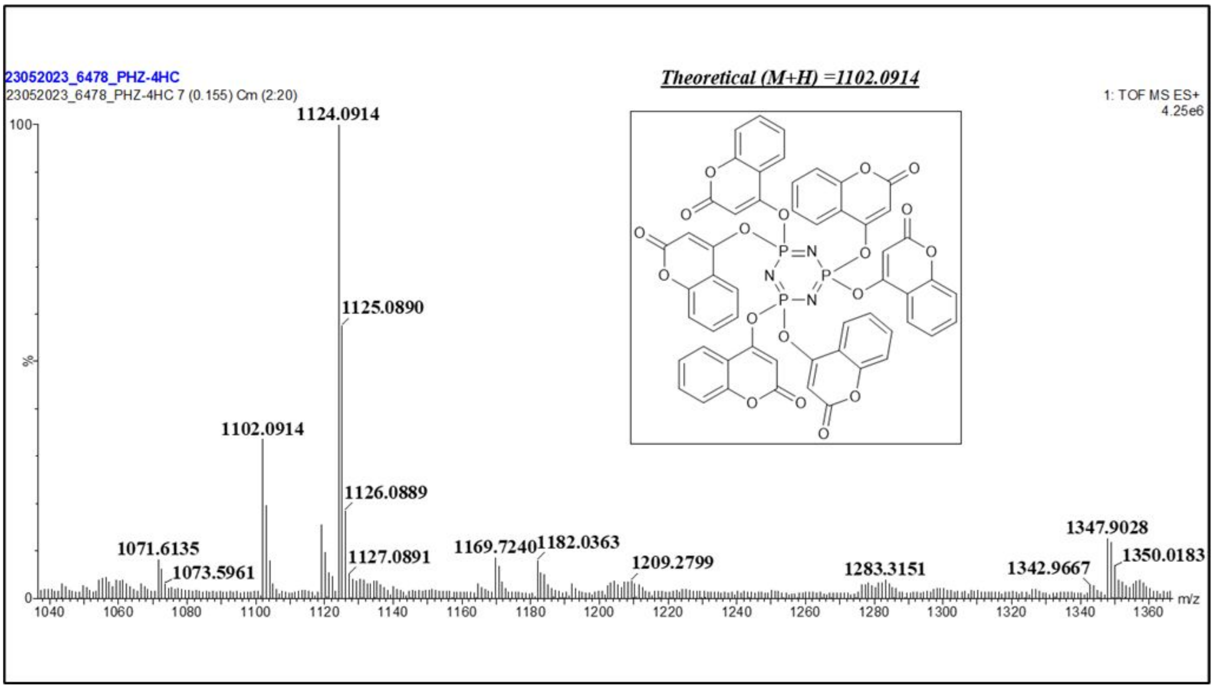

MS (ESI-MS), *m/z*: calc. for $C_{54}H_{30}N_3O_{18}P_3$ 1102; found 1124 $[M+Na]^+$

Fig. S5. HRMS spectrum of **Cpz-4-HC**

4. U.V-VIS spectroscopy

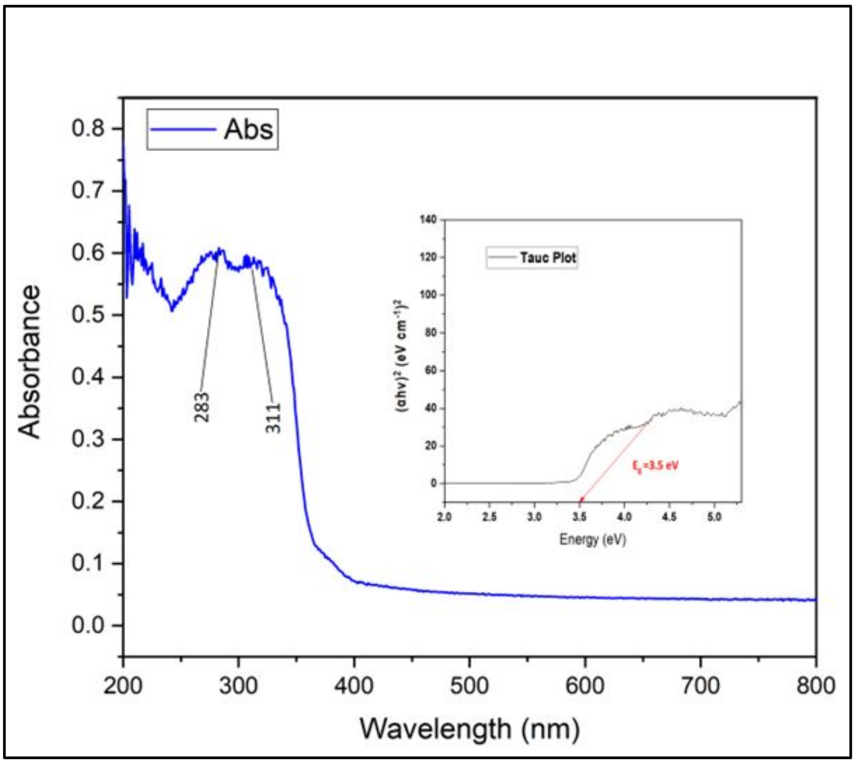

Fig. S6. Solid state absorption spectrum of **Cpz-4-HC**.

5. Fluorescence spectroscopy

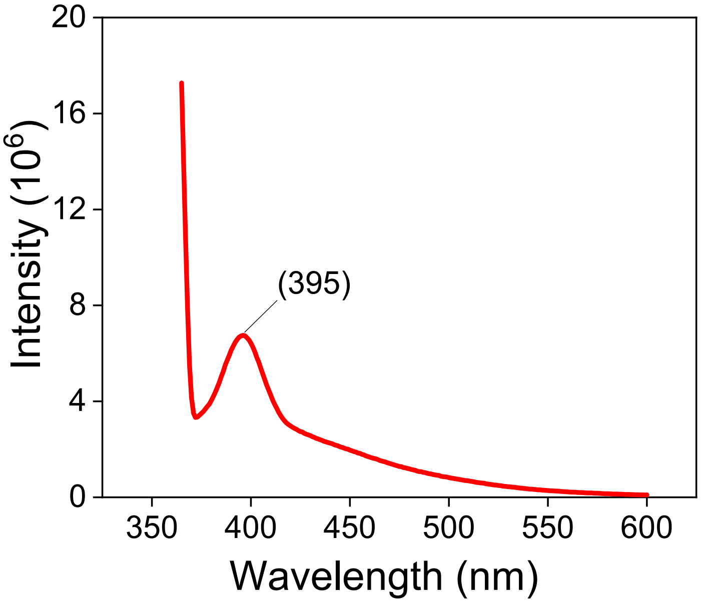

Fig. S7. Fluorescence spectra of **Cpz-4-HC**

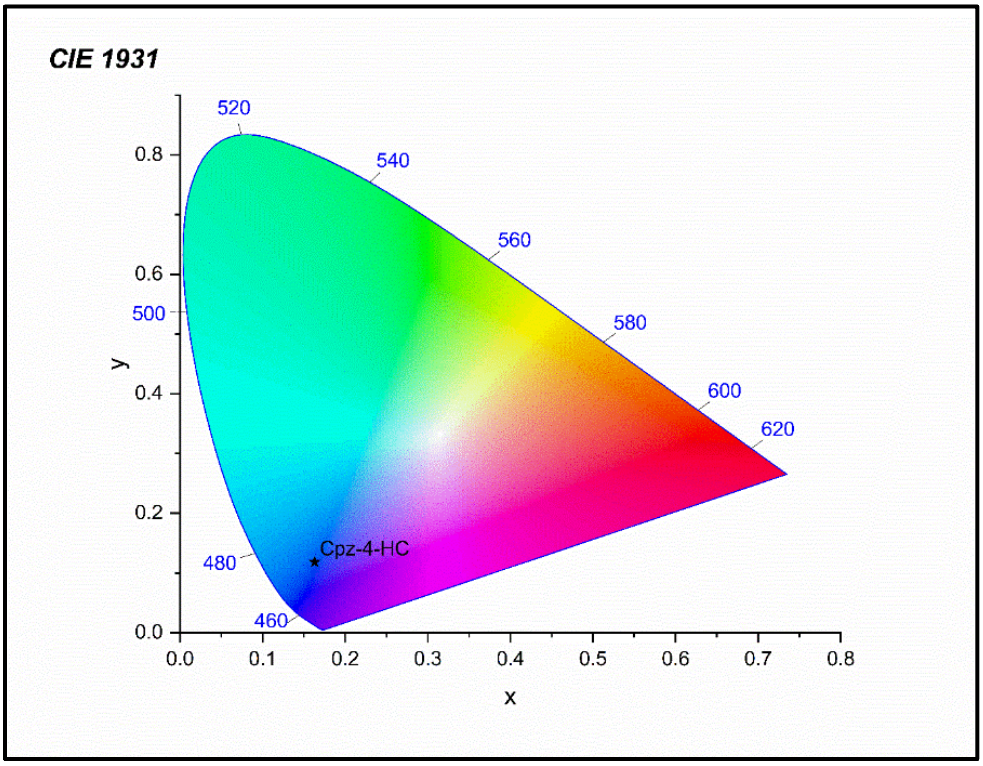

Fig. S8. The CIE 1931 plot of synthesized Cpz-4-HC with blue emission with coordinates (x= 0.163013376 and y=0.118349325).

Section S-2: Selective detection of TNP by Cpz-4-HC

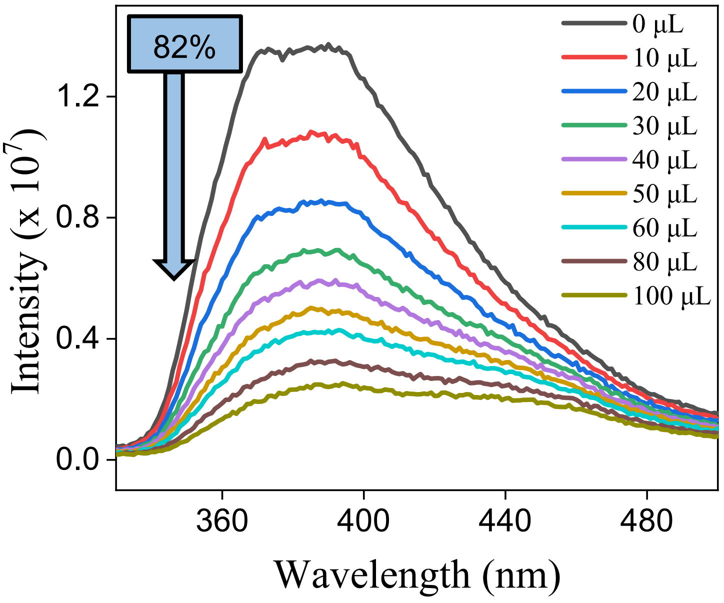

Fig. S9. Emission spectrum of **Cpz-4-HC** dispersed in water after adding aqueous 2,4-DNP solution.

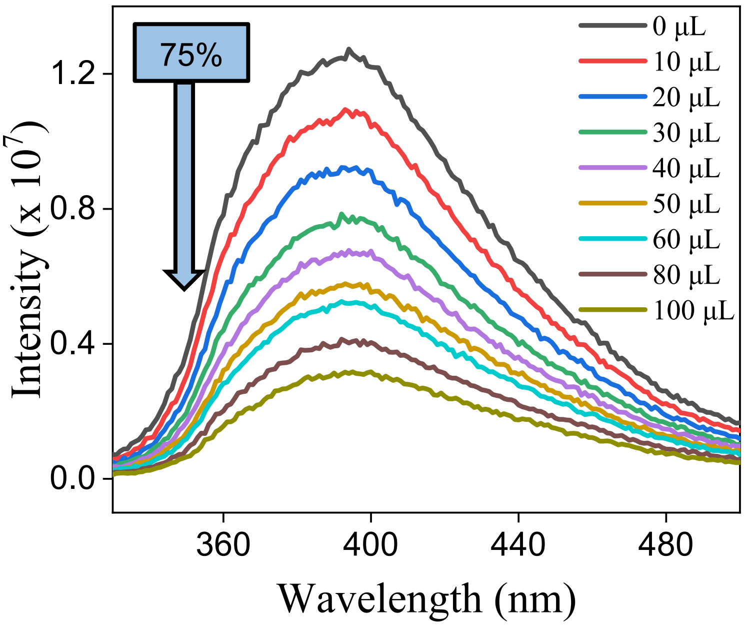

Fig. S10. Emission spectrum of **Cpz-4-HC** dispersed in water after adding aqueous 4-NP solution.

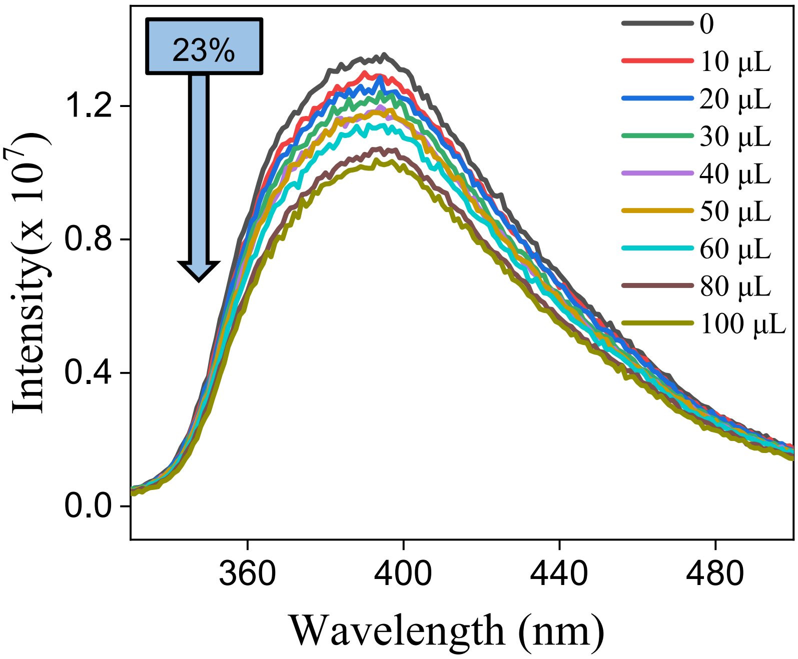

Fig. S11. Emission spectrum of **Cpz-4-HC** dispersed in water after adding aqueous 2-NP solution.

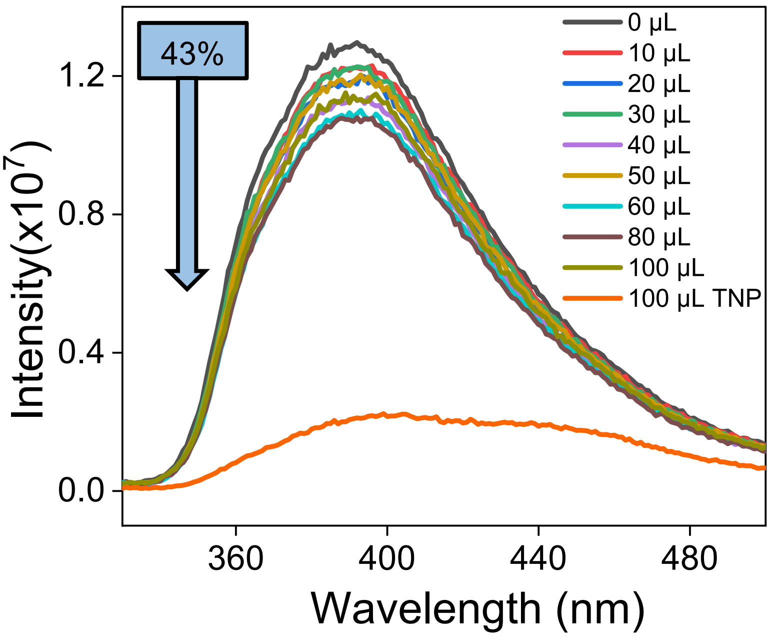

Fig. S12. Emission spectrum of **Cpz-4-HC** dispersed in water after adding aqueous 1,3-DNB solution.

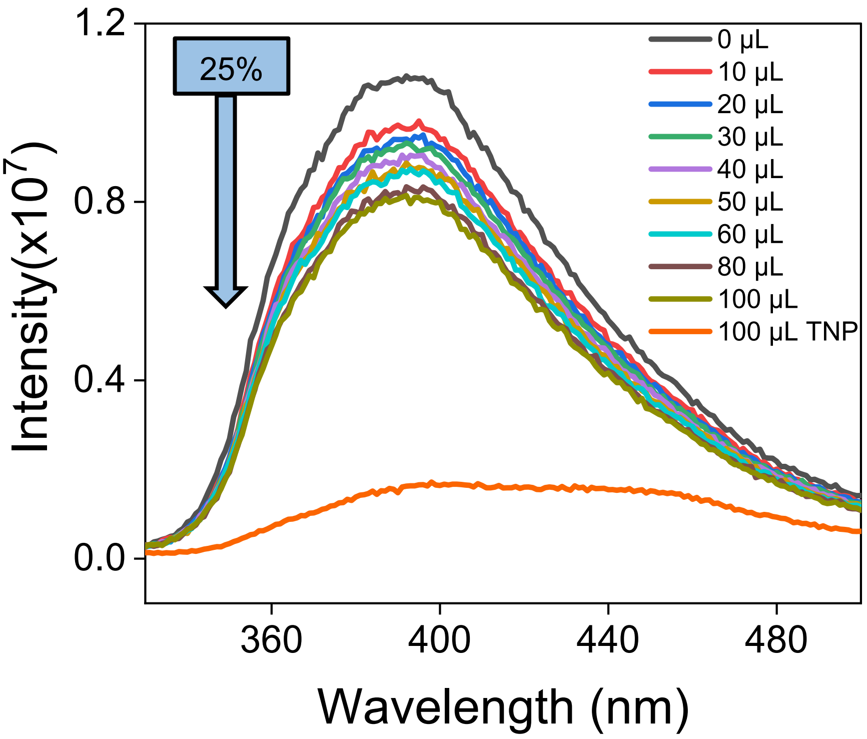

Fig. S13. Emission spectrum of **Cpz-4-HC** dispersed in water after adding aqueous 2,4-DNT solution.

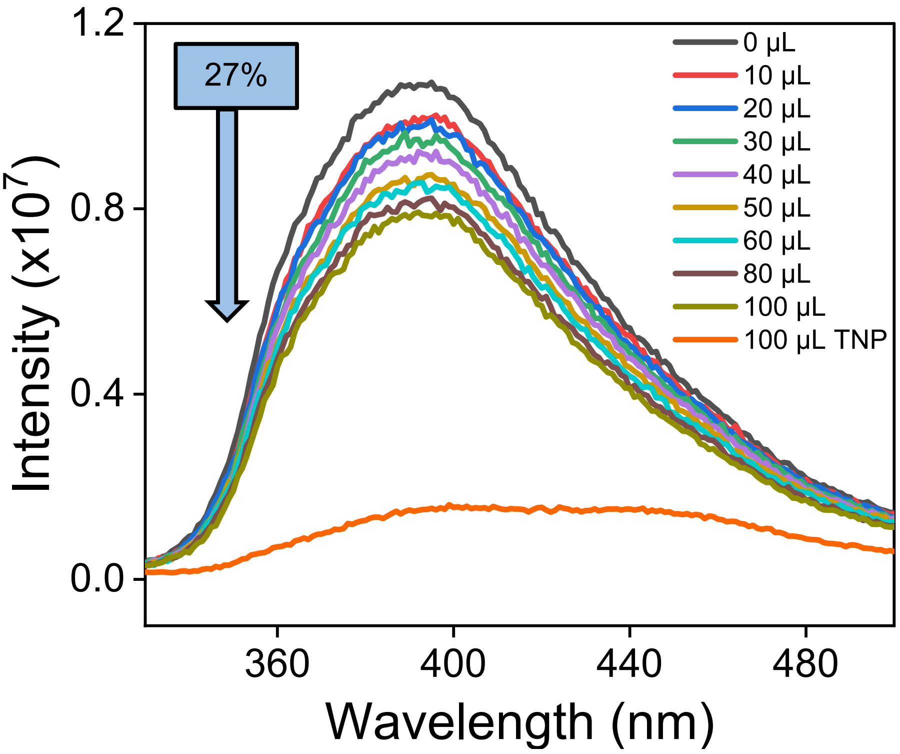

Fig. S14. Emission spectrum of **Cpz-4-HC** dispersed in water after adding aqueous 2,6-DNT solution.

Section S-3: Calculation of detection of limit

Table S1. Calculation standard deviation and detection limit

Blank Readings (Cpz-4-HC)	FL Intensity Reading
**Reading 1**	1.43E+07
**Reading 2**	1.42E+07
**Reading 3**	1.42E+07
**Reading 4**	1.44E+07
**Standard Deviation (σ)**	95742.71

Slope from Graph (m)	1.97E+11
**Detection limit (3σ/m)**	**0.334 ppm**

Section S-4: Stern-Volmer plot of Cpz-4-HC.

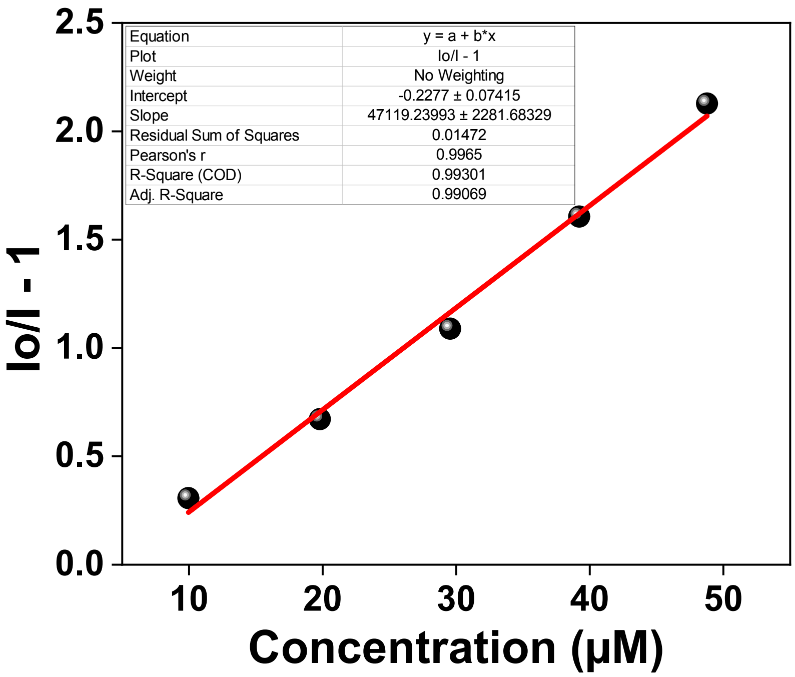

Figure S15. Stern-Volmer plot for TNP. The relative fluorescence intensity is linear with TNP concentration in the range of 0 – 50 µM, $I_0/I = 1 + 47119.23$ ([TNP]) ($R^2 =$ 0.99069).

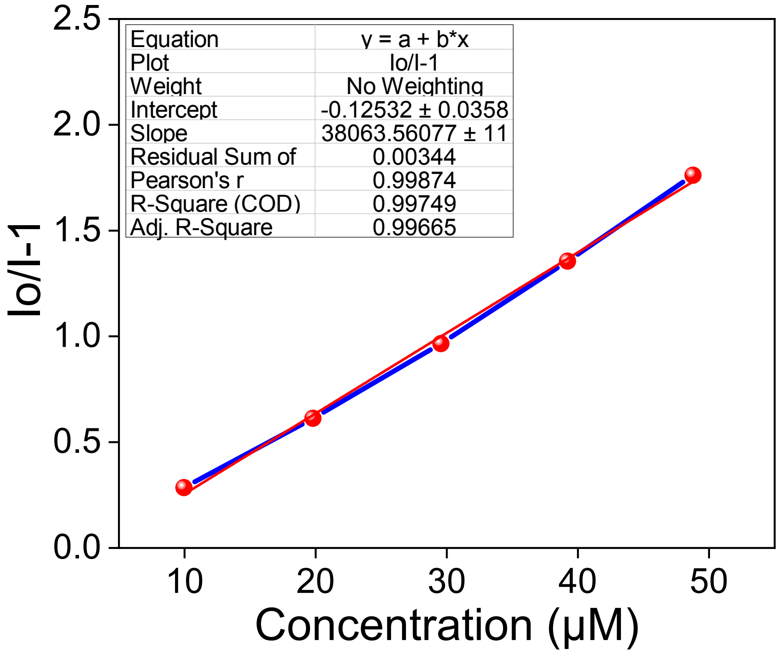

Figure S16. Stern-Volmer plot for 2,4-DNP. The relative fluorescence intensity is linear with 2,4-DNP concentration, $I_0/I = 1 + 38063.56$ ([2,4-DNP]) ($R^2 = 0.996$).

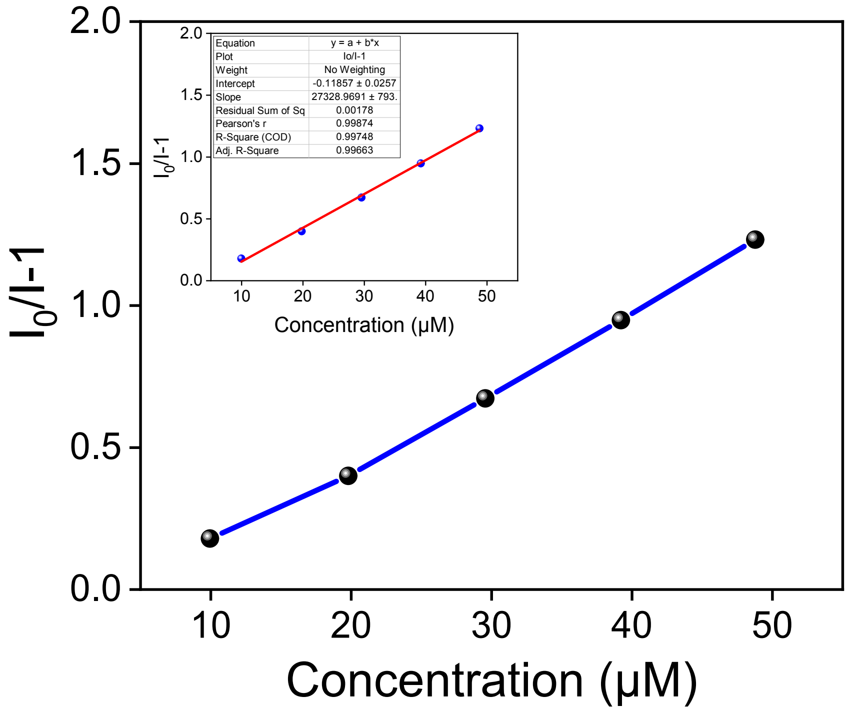

Figure S17. Stern-Volmer plot for 4-NP. The relative fluorescence intensity is linear with 4-NP concentration, $I_0/I = 1 + 27328.96$ ([4-NP]) ($R^2 = 0.996$).

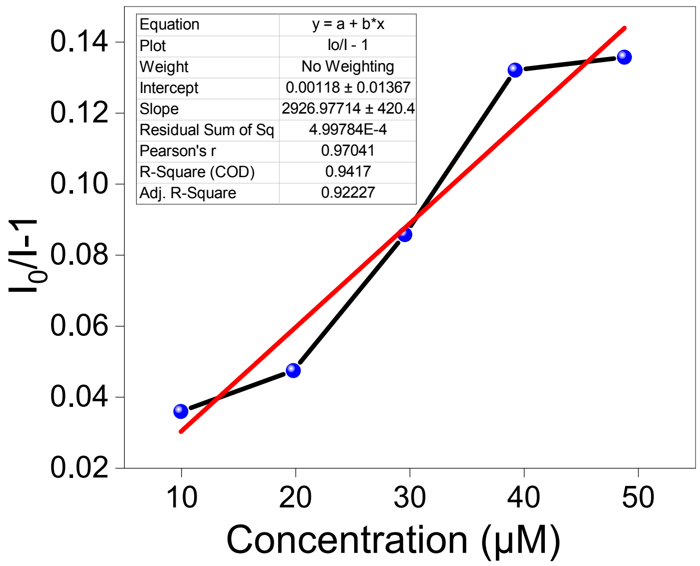

Figure S18. Stern-Volmer plot for 2-NP. The relative fluorescence intensity is linear with 2-NP concentration, $I_0/I = 1 + 2926.97$ ([2-NP]) ($R^2 = 0.922$).

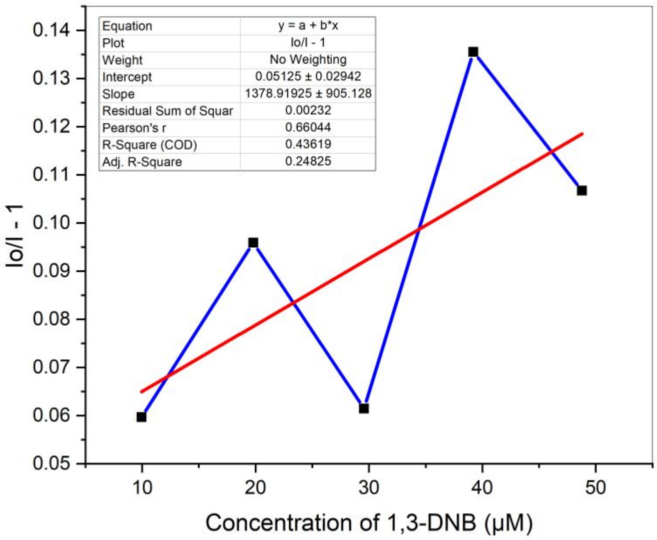

Figure S19. Stern-Volmer plot for 1,3-DNB. The relative fluorescence intensity is linear with 1,3-DNB concentration, $I_0/I = 1 + 1378.91([1,3\text{-DNB}])$ (R^2 =0.24825).

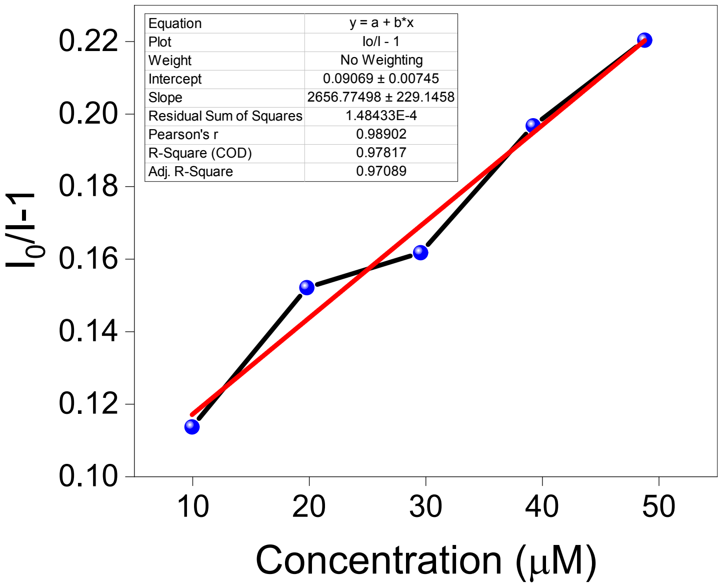

Figure S20. Stern-Volmer plot for 2,4-DNT. The relative fluorescence intensity is linear with 2,4-DNT concentration, $I_0/I = 1 + 2656.774([2,4\text{-DNT}])$ (R^2 =0.97089).

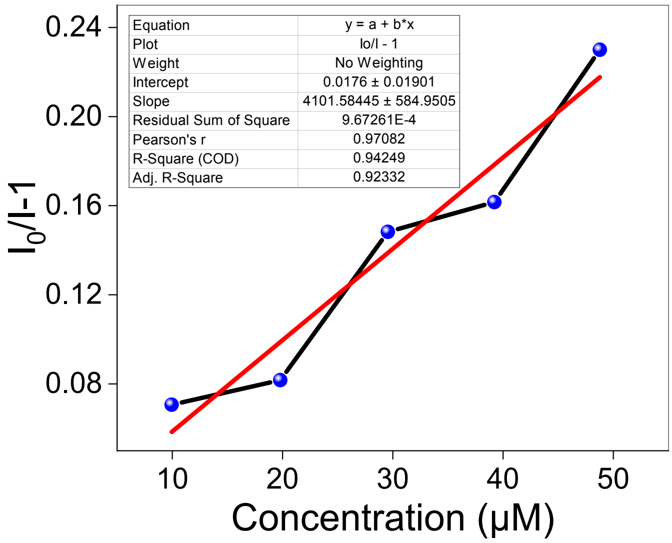

Figure S21. Stern-Volmer plot for 2,6-DNT. The relative fluorescence intensity is linear with 2,6-DNT concentration, $I_0/I = 1 + 4101.584([2,6\text{-DNT}])$ (R^2 =0.92332).

Table S2: Comparison of **Cpz-4-HC** with other reported TNP sensor

Sensor	K_{sv} (M^{-1})	Detection Limit	Medium	Ref.
DAAQ	3.67 x 10^4	8.19 µM	DMSO	[1]
1	1.31 x 10^4	5.93 µM	water	[2]
{[Zn(mbhna)(bpma)]}$_n$<br>{[Cd(mbhna)(bpma)]·DMF}$_n$<br>{[Zn(mbhna)(bpea)]}$_n$<br>{[Cd(mbhna)(bpea)]}$_n$<br>{[Zn(mbhna)(bpta)]}$_n$<br>{[Cd(mbhna)(bpta)(CH_3OH)]}$_n$<br>{[Zn(mbhna)(bpba)]·CH_3OH·H_2O}$_n$<br>{[Cd(mbhna)(bpba)]}$_n$	7.80 x 10^3<br>1.09 x 10^4<br>1.17 x 10^4<br>8.90 x 10^3<br>1.70 x 10^4<br>1.60 x 10^4<br>1.44 x 10^4<br>1.17 x 10^4	1.35 ppm<br>0.525 ppm<br>0.595 ppm<br>0.642 ppm<br>1.1 ppm<br>0.471 ppm<br>0.394 ppm<br>0.240 ppm	water<br>water<br>water<br>water<br>water<br>water<br>water<br>water	[3]
DMANSOC	NA	4.64 µM	methanol	[4]
[Zn_4($BDCPPI)_2$($DMF)_6$]·3DMF·4H_2O (1)<br>[Cu_2(BDCPPI)($H_2O)_2$]·5DMF·6H_2O (2)<br>[Cd_4($BDCPPI)_2$($DMF)_2$($H_2O)_3$]·2DMF·8H_2O (3)	6.72×10^4<br>2.22×10^4<br>3.66×10^4	0.89 µM<br>2.70 µM<br>1.63 µM	water<br>water<br>water	[5]
Co-MOF<br>Zn-MOF	3.73×10^4<br>3.35×104	1.60 µM<br>1.79 µM	ethanol<br>ethanol	[6]
[Cd(L1)($NCS)_2$$H_2O$]$_n$<br>[$Cd_{1.5}$(L1)($N(CN)_2)_3$]$_n$<br>[Cd(L2)($NCS)_2$]$_n$<br>[$Cd_{1.5}$(L2)($N(CN)_2)_3$]$_n$	3.4×10^4<br>6.9×10^4<br>2.3×10^4<br>2.3×10^4	55 µM<br>28 µM<br>27 µM<br>31 µM	acetonitrile<br>acetonitrile<br>acetonitrile<br>acetonitrile	[7]
1@H_4TBAPy<br>complex 1	3.16 × 10^4<br>1.84 × 10^4	1.71µM<br>6.08 µM	ethanol<br>ethanol	[8]
DLC	2.17 x 10^4	4.3 µM	acetonitrile	[9]
Cu(I)-MOF-7<br>Cu(I)-MOF-8<br>Cu(I)-MOF-9<br>Cu(I)-MOF-10	3.9 × 10^4<br>3.67 × 10^4<br>4.53 × 10^4<br>6.93 × 10^4	2.99 × 10^{-6}M (2.99ppm)<br>2.76 × 10^{-6}M (2.76ppm)<br>1.21 × 10^{-6}M (1.21ppm)<br>2.47 × 10^{-6}M (2.47ppm)	DMSO<br>DMSO<br>DMSO<br>DMSO	[10]
sensor L	2.44 × 10^4	9.17 × 10^{-7} M	acetonitrile	[11]
Py-HP CHOF	NA	0.41 µM	ethanol	[12]
H_2L	2.25 × 10^5	6 × 10^{-8} M	DMSO–H_2O (9 : 1)	[13]
{[Co(TPTC)($HBPDPE)_2$·2H_2O]·3H_2O}$_n$	2.08 × 10^5	NA	DMF	[14]
[Cd_2L($HBTC)_2$·2H_2O·2i-PrOH]$_n$	3.01 × 10^4	7.31x10^{-2}mM	water	[15]
**Cpz-4-HC**	**4.71× 10^4**	**0.334 ppm**	**Water**	**This Work**

Table S3. Summary of Stern-Volmer rate constant (K_{sv}) of **Cpz-4-HC** for different NACs at room temperature.

Nitro-phenols	S-V Constant (K_{sv}) M^{-1}
**2,4,6-TNP**	47119.23
**2,4-DNP**	38063.56
**4-NP**	27328.96
**2-NP**	2926.97
**1,3-DNB**	1378.91
**2,4-DNT**	2656.774
**2,6-DNT**	4101.584

References:

[1] S. Gadiyaram, A. Ghosh, V. D. Ghule, P. K. Sharma, and D. Amilan Jose, ‘Aggregation-induced emission active multianalyte sensor: Detection of pH, carbonate, bi-carbonate and nitroaromatics in water’, *Microchemical Journal*, vol. 204, p. 110957, 2024, doi: https://doi.org/10.1016/j.microc.2024.110957.

[2] Y. Meng, Y. Cheng, X. Yang, X. Lv, X. Huang, and D. Schipper, ‘Rapid and reliable ratiometric fluorescence detection of nitro explosive 2,4,6-trinitrophenol based on a near infrared (NIR) luminescent Zn(II)-Nd(III) nanoring’, *Spectrochim Acta A Mol Biomol Spectrosc*, vol. 318, p. 124468, 2024, doi: https://doi.org/10.1016/j.saa.2024.124468.

[3] A. Chanda and S. K. Mandal, ‘Selective and ultrafast sensing of 2,4,6-trinitrophenol - A nitro-explosive and mutagenic pollutant - In aqueous media by highly stable and recyclable metal-organic probes: Design principles and mechanistic studies’, *Dyes and Pigments*, vol. 210, p. 111025, 2023, doi: https://doi.org/10.1016/j.dyepig.2022.111025.

[4] V. Desai *et al.*, ‘Design and Synthesis of an Efficient Fluorescent Probe Based on Oxacalix[4]arene for the Selective Detection of Trinitrophenol (TNP) Explosives in Aqueous System’, *J Fluoresc*, vol. 34, no. 3, pp. 1219–1228, 2024, doi: 10.1007/s10895-023-03352-7.

[5] Y. Xiao *et al.*, ‘Aromatimide tetracarboxylate metal-framework materials: Fluorescence sensing application towards NACs and small drug molecules’, *J Mol Struct*, vol. 1310, p. 138362, 2024, doi: https://doi.org/10.1016/j.molstruc.2024.138362.

[6] T. Verma, U. P. Singh, P. Verma, R. J. Butcher, C. Ghosh, and P. Roy, ‘Synthesis of luminescent naphthalene diimide based nano Co/Zn organic frameworks: Cell imaging, sensing studies of explosive compound and pesticide’, *J Mol Struct*, vol. 1302, p. 137467, 2024, doi: https://doi.org/10.1016/j.molstruc.2023.137467.

[7] J. Mandal *et al.*, ‘Chromone-Based Cd(II) Fluorescent Coordination Polymer Fabricated to Study Optoelectronic and Explosive Sensing Properties’, *Inorg Chem*, vol. 63, no. 10, pp. 4527–4544, Mar. 2024, doi: 10.1021/acs.inorgchem.3c03646.

[8] R. Huo, T. Zhang, G. Zeng, C. Wang, Y. H. Xing, and F. Y. Bai, ‘Construction of Naphthalenediimide Lanthanide(III)-MOFs and Composites Incorporated Electron-Rich Pyrene Derivative as Multifunctional Fluorescence Sensing for Nitro Aromatic Compounds and Aldehydes’, *Chin J Chem*, vol. 42, no. 3, pp. 283–293, Feb. 2024, doi: https://doi.org/10.1002/cjoc.202300448.

[9] S. Gadiyaram, M. Aakshika Sree, N. Sharma, and D. Amilan Jose, ‘An amphiphilic dansyl based multianalyte sensor for the detection of Hg2+, PPi, and TNP: A three-in-one chemical sensor’, *Methods*, vol. 223, pp. 45–55, 2024, doi: https://doi.org/10.1016/j.ymeth.2024.01.007.

[10] X. Xiao *et al.*, ‘Crystal structures and syntheses of four novel Cu(I)-MOFs with in-situ reduced of Cu(II) and highly efficient fluorescent sensor for trace nitroaromatics’, *J Mol Struct*, vol. 1300, p. 137311, 2024, doi: https://doi.org/10.1016/j.molstruc.2023.137311.

[11] Y.-L. Liu *et al.*, ‘A facile and sensitive hexahomotrioxacalix[3]arene-based fluorescent sensor for the detection of trace amounts of 2,4,6-trinitrophenol’, *Science of The Total Environment*, vol. 908, p. 168209, 2024, doi: https://doi.org/10.1016/j.scitotenv.2023.168209.

[12] T. Huo and Y. He, ‘Novel Covalent Bonds and Hydrogen Bonds Linked Porous Organic Frameworks as Chemosensor for Detecting 2,4,6-Trinitrophenol in Water and Soil Samples’, *ACS Appl Mater Interfaces*, vol. 16, no. 16, pp. 21233–21241, Apr. 2024, doi: 10.1021/acsami.4c03375.

[13] P. Das, M. Das, R. Biswas, S. Laha, B. C. Samanta, and T. Maity, ‘Morphological adaptability through structural alterations in an AIE active novel chemosensor with Al(iii), Fe(iii), and gas phase/aqueous phase TNP recognition ability’, *New Journal of Chemistry*, vol. 48, no. 13, pp. 5820–5833, 2024, doi: 10.1039/D4NJ00303A.

[14] C. Wang, W. Sheng, C. Sun, J. Lei, and J. Hu, ‘A cobalt-coordination polymer as a highly selective and sensitive luminescent sensor for detecting 2,4,6-trinitrophenol’, *Molecular Crystals and Liquid Crystals*, vol. 768, no. 3, pp. 117–126, Feb. 2024, doi: 10.1080/15421406.2023.2262857.

[15] J. Zhang, Z. Lin, Y. Yue, Q. Chen, D. Yin, and C. Zhang, ‘A photochromic Cd(ii)-organic framework showing highly efficient dual-response sensing properties’, *New Journal of Chemistry*, vol. 47, no. 47, pp. 21986–21993, 2023, doi: 10.1039/D3NJ04122K.
